# Identification and classification of papain-like cysteine proteinases

**DOI:** 10.1016/j.jbc.2023.104801

**Published:** 2023-05-08

**Authors:** Fatih Ozhelvaci, Kamil Steczkiewicz

**Affiliations:** Institute of Biochemistry and Biophysics, Polish Academy of Sciences, Warsaw, Poland

**Keywords:** cysteine protease, deubiquitylation, enzyme structure, homology modeling, peptidase, protease, protein function and prediction

## Abstract

Papain-like cysteine peptidases form a big and highly diverse superfamily of proteins involved in many important biological functions, such as protein turnover, deubiquitination, tissue remodeling, blood clotting, virulence, defense, and cell wall remodeling. High sequence and structure diversity observed within these proteins hinders their comprehensive classification as well as the identification of new representatives. Moreover, in general protein databases, many families already classified as papain like lack details regarding their mechanism of action or biological function. Here, we use transitive remote homology searches and 3D modeling to newly classify 21 families to the papain-like cysteine peptidase superfamily. We attempt to predict their biological function and provide structural characterization of 89 protein clusters defined based on sequence similarity altogether spanning 106 papain-like families. Moreover, we systematically discuss observed diversity in sequences, structures, and catalytic sites. Eventually, we expand the list of human papain–related proteins by seven representatives, including dopamine receptor–interacting protein 1 as potential deubiquitinase, and centriole duplication regulating CEP76 as retaining catalytically active peptidase-like domain. The presented results not only provide structure-based rationales to already existing peptidase databases but also may inspire further experimental research focused on peptidase-related biological processes.

Peptidases constitute a class of enzymes catalyzing peptide bond breakage in either proteins or other biologically relevant molecules. They are essentially indispensable for protein turnover, digestion, signaling, tissue remodeling, and many more. Peptidases adopt two major catalytic mechanisms involving a water molecule or hydrophilic residue to attack the peptide bond. Hence, the most general classification of peptidases is based on the very catalytic residue type: cysteine, serine, threonine, aspartate, and glutamate. A separate class of peptidases utilize metal ions for catalysis; those define a separate clade named metalloproteases. This classification has been detailed at the level of protein families and clans and compiled into today's peptidase reference, the MEROPS database. The currently known peptidase universe is organized according to the detectable similarity in sequence, and function, as well as based on experimental data.

Each peptidase catalytic type may be scaffolded by a range of structural folds. For instance, cysteine peptidases may retain cysteine proteinase, caspase-like, sortase, eIF1-like, Ntn hydrolase–like, or trypsin-like serine protease folds. In other words, they may belong to multiple protein superfamilies. Furthermore, each peptidase-related superfamily may cover many protein families, which is especially the case for cysteine proteinases, zincin-like metallopeptidases, and trypsin-like serine proteases. On the other hand, the given superfamily may also group peptidases of multiple catalytic types, like trypsin-like proteases (cysteine and serine type) and Ntn hydrolase–like peptidases (cysteine, serine, and threonine), which might indicate a convergent evolution of catalytic sites. For such broader superfamilies, despite provided clear-cut classifications, it is still challenging to fully comprehend their diversity and rationally describe it in the light of performed biological functions.

Papain-like cysteine proteinases, a subclass of cysteine peptidases, constitute a large and diverse superfamily of proteins from all kingdoms of life, including many viruses (1, 2). Papain, the archetypal member of the superfamily, is a protease extracted from the papaya plant used in native Indian countries for tendering uncooked meat, and it was one of the first discovered enzymes (3). Papain-like enzymes act as endopeptidases, dipeptidases, and exopeptidases (carboxypeptidases and aminopeptidases) or cleave amide groups outside the protein's main chain, for example, glutaminases, acyltransferases, and peptidoglycan amidases. They may be secreted to play roles as tissue remodelers (4) or virulence/defense factors (5, 6) or function as deubiquitinases important for multiple regulatory pathways (7). They function as accessory peptidases in multiple viruses (8, 9, 10) and are used as toxins by many bacterial species (11, 12, 13, 14). Some families also underwent significant expansions, for example, deubiquitinating enzymes, with paralogs highly specialized for particular substrates (15, 16).

Papain-like cysteine proteases share a common structural core classified in the SCOP database (17) as an α + β fold (d.3—cysteine proteinases) comprising an α-helix followed by antiparallel β-sheet composed of four/five β-strands (depending on database version) with 43215 topology (Fig. 1). The active site is located between the helix providing the catalytic cysteine residue located at its N terminus and β-strands 2 and 3 delivering histidine and a polar group, respectively. These two strands are also slightly bent away from each other creating a convenient pocket for aligning the incoming substrate for catalysis.

**Figure 1:**
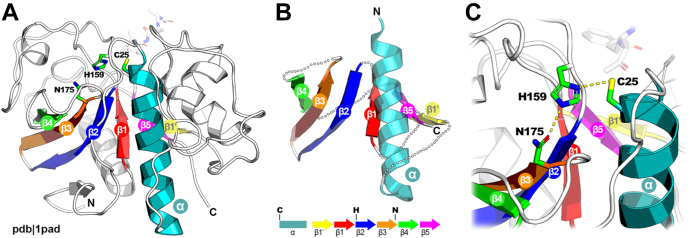
**Structural core of papain-like enzymes.***A*, the first crystallographic structure solved for papain enzyme from *Carica papaya* (287), secondary structure elements defining the core are rendered in colors whereas remaining parts of the structure are bleached. *B*, structural core of papain-like cysteine proteinase distilled from original Protein Data Bank structure, noncore parts of the structure were omitted for clarity; below, the reference topology with catalytic residues marked above corresponding positions. *C*, catalytic site of papain.

A minimal canonical catalytic site of cysteine proteinases is formed by a cysteine–histidine dyad (18, 19). The thiol group of the cysteine acts as a nucleophile, whereas histidine acts as a base and forms reactive thiolate–imidazolium ion pair during the catalysis (20), although in some known proteins, the distance between histidine and cysteine is too big, which limits this effect suggesting that nucleophilicity of the cysteine, located at the N terminus of α-helix, might be primarily increased by α-helix dipole effect itself (21). Still, the majority of cysteine proteinases deploy a catalytic triad with additional polar residue orienting histidine's imidazole ring toward cysteine and increasing its basicity (22).

The topology of the dyad–triad is conserved across the superfamily with only a few exceptions known from structural studies. For instance, GP42 transglutaminase from *Phytophthora sojae* lacks histidine at its canonical position—this residue is located immediately after the cysteine on α-helix and forms a salt bridge with aspartate from another nearby α-helix (23); human Atg4b has its histidine located at third β-strand, two positions before aspartate (24); transglutaminase from *Streptomyces mobaraense* has histidine and aspartate residues swapped (25).

The observed abundance of functions, specificities, taxonomic distributions, and ecological contexts entails a great diversity in structures and sequences of papain-like peptidases. Despite sharing a common structural core, these proteins display a remarkably rich repertoire of insertions (*e.g.*, the “fingers” domain of deubiquitinases (26)), deletions (*e.g.*, viral accessory proteins (27)), permutations (28, 29, 30), and overall spatial deteriorations. This hinders the classification and identification of new representatives of this superfamily, even at the level of their structures, which tend to display diversity surpassing the capabilities of automated structure comparison approaches. This project aims to collect and classify sequences and structures of all known cysteine proteinases, distill a set of features describing these proteins, and identify new superfamily members.

SCOP database assigns 24 families to the “cysteine proteinases” superfamily (d.3.1), whereas Pfam, a protein family database, classifies 78 protein families into the Peptidase_CA clan (CL0125), including proteases, acetyltransferases, transglutaminases, deubiquitinases, amidases, and multiple domains of unknown function (DUFs). MEROPS defines five clans (CA, CE, CN, CO, and CP) spanning 54 families crosslinked to 40 Pfam families from the Peptidase_CA clan. In addition, five more MEROPS families, that is, C7, C23, C27, C36, and C42, remain unassigned to any MEROPS clan despite linking to the aforementioned Pfam clan.

There have been few reviews discussing papain-like families to date. In 1994, Rawlings and Barrett (31) identified 21 cysteine peptidase families, of which, according to the authors, three were related to papain (C1, C2, and C10). From the current point of view, that dataset included 12 papain-like, three caspase-like, and two trypsin-like families. They also recognized that cysteine peptidases may differ in His–Cys order within amino acid sequences; however, because of the lack of structural data, it was hardly possible to recognize circular permutations at that time. A year later, they classified 35 peptidase families into five clans: CA, CB, CC, CD, and CE (32), and the next follow-up article covered seven clans and 48 MEROPS families, of which two clans and 27 families were related to papain (18). In 1999, Koonin's group (33) described a novel papain-like superfamily of transglutaminases containing seven protein families; according to the current Pfam database, five of those families are now included in Transglut_core PF01841 (families numbered 1, 4, 5, 6, and 7 in the original article), one in DUF553 PF04473 (no. 3), and one family has not been defined in Pfam yet (no. 2 therein). Further work on the identification and classification of papain-like enzymes was done by Aravind *et al.* (30) who described NlpC/P60 superfamily as related to papain. The report included four major protein families: NLPC_P60, CHAP, C92, and lecithin retinol acyltransferase (LRAT) and systematically discussed circular permutations and evolutionary relationships within the superfamily. Furthermore, based on sequence similarity searches and biological context analyses, PPPDE proteins could be identified as papain-like enzymes (28).

Eventually, two major works aimed to provide a general view of the evolutionary history of the peptidase universe. In 2007, Di Cera *et al.* (1) analyzed the distribution of MEROPS families within sequenced genomes to identify sets of ubiquitous (16 families) and kingdom-specific sets of peptidases. Similarly, in 2019, Rawlings and Bateman (2) surveyed the distribution of 271 peptidase families across the tree of life and found 33 to be common to all cellular organisms.

There has been neither comprehensive analysis of papain-like cysteine peptidase diversity, which would discuss neither complex sequence-to-structure-to-function relationships nor systematic searches for new superfamily members. The aim of this report is to identify new members of papain-like superfamily as well as to categorize the diversity of these proteins. By using sequence-based remote homology detection methods, we newly classify 28 Pfam and three MEROPS families, seven human proteins, as well as 16 Protein Data Bank representatives (PDB90) not yet belonging to any family as papain-like. Based on manually curated superimpositions, we provide structure-based multiple sequence alignments for the first time outlining the most complete view on diversity within all known families retaining cysteine proteinase fold.

## Results

### Identification of new families

**Figure 2:**
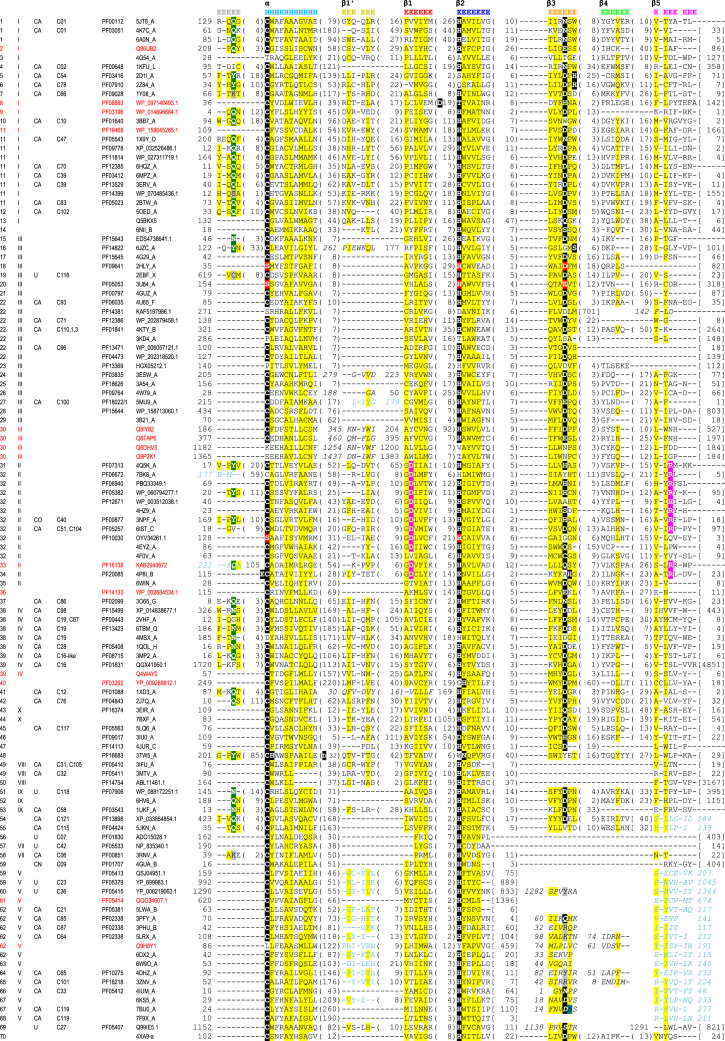

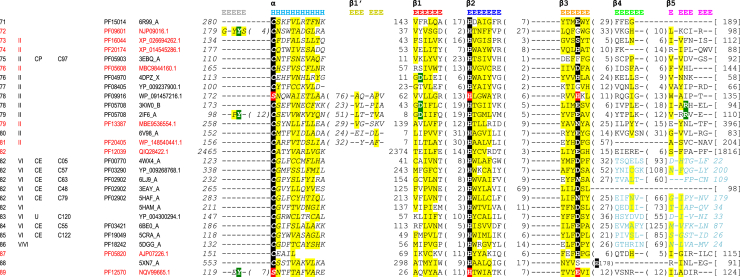
**Structure-guided multiple sequence alignment of structural core elements for proteins representing all identified papain-like families.** The *columns* from the *left*: group number, cluster number, MEROPS clan, MEROPS family, Pfam family, representative protein identifier—either National Center for Biotechnology Information GenBank entry or Protein Data Bank code. Tags for families newly classified as papain-like in this article are written in *red font*. The numbers within the alignment area denote residues omitted from the alignment: in *parentheses*—insertions between two elements; in s*quare brackets*—C-terminal part; *without any brackets*—the number of the first residue in the following block. Sequences written in *italic* indicate permuted elements, whereas sequences in *turquoise* are written in reverse and correspond to structural elements oriented in a reverse direction in 3D space; these sequences are followed by their first residue number. Sequence conservation is marked with highlights as follows: *yellow*—nonpolar, *gray*—charged, *black/green/red*—confirmed or predicted active-site residues (*red* for proteins with serine instead of cysteine), *magenta*—Asp/Arg residues characteristic for NlpC/P60-like proteins.

Starting from the initial set of 78 Pfam and 59 MEROPS families of papain-like proteins, we identified additional 28 Pfam and 30 MEROPS families, 25 Clusters of Orthologous Groups of proteins (COG) and 47 EuKaryotic Orthologous Group (KOG) families, as well as 21 PDB90 structures and seven human proteins not belonging to any Pfam family (Table S1). The dataset counts over 4.8 million nonredundant protein sequences, 2.2 million of which belong to single family PF08715 of coronavirus papain-like peptidases. Markov clustering (MCL) algorithm based on hmmscan scores defined 89 groups of closely related families represented by Pfam, PDB90, COG/KOG, MEROPS, or human proteins (Fig. 2 and Table S1). Further clustering based on HHsearch scores that reflect more distant similarities allowed us to define 10 bigger “meta” groups, each containing at least two Pfam families, which share multiple structural features.

Despite being assigned to the Pfam CL0125 clan, eight protein families were still annotated as DUFs with no additional information regarding their probable biological roles and sequence–structure alignment to already known papain-like domains. Peptidase family C8 (PF03569) belonging to the CL0125 Pfam clan could not be confirmed as papain-like according to the methods used. Moreover, two of the identified families, PF18021 and PF18022, were assigned to the Trefoil clan (CL0066), probably according to the beta-trefoil lectin-like domain preceding the peptidase domain—here, we classify them as papain-like.

Eventually, 16 Pfam families, 13 of which retain conserved catalytic residues, had not been previously classified or annotated as related to papain-like cysteine peptidases. Seven human proteins: BIVM (Swiss-Prot [SP]: Q86UB2, basic immunoglobulin-like variable motif-containing protein), DRC7 (SP: Q8IY82, dynein regulatory complex subunit 7), CEP76 (SP: Q8TAP6, centrosomal protein), C2D2B (SP: Q6DHV5), C2D2A (SP: Q9P2K1), C14orf28 (SP: Q4W4Y0), VRTN (SP: Q9H8Y1, vertnin), of which only three lack potential catalytic sites (C2D2A, C2D2B, and vertnin), had also not been previously recognized to contain papain-like domains.

### Structural diversity

**Figure 3:**
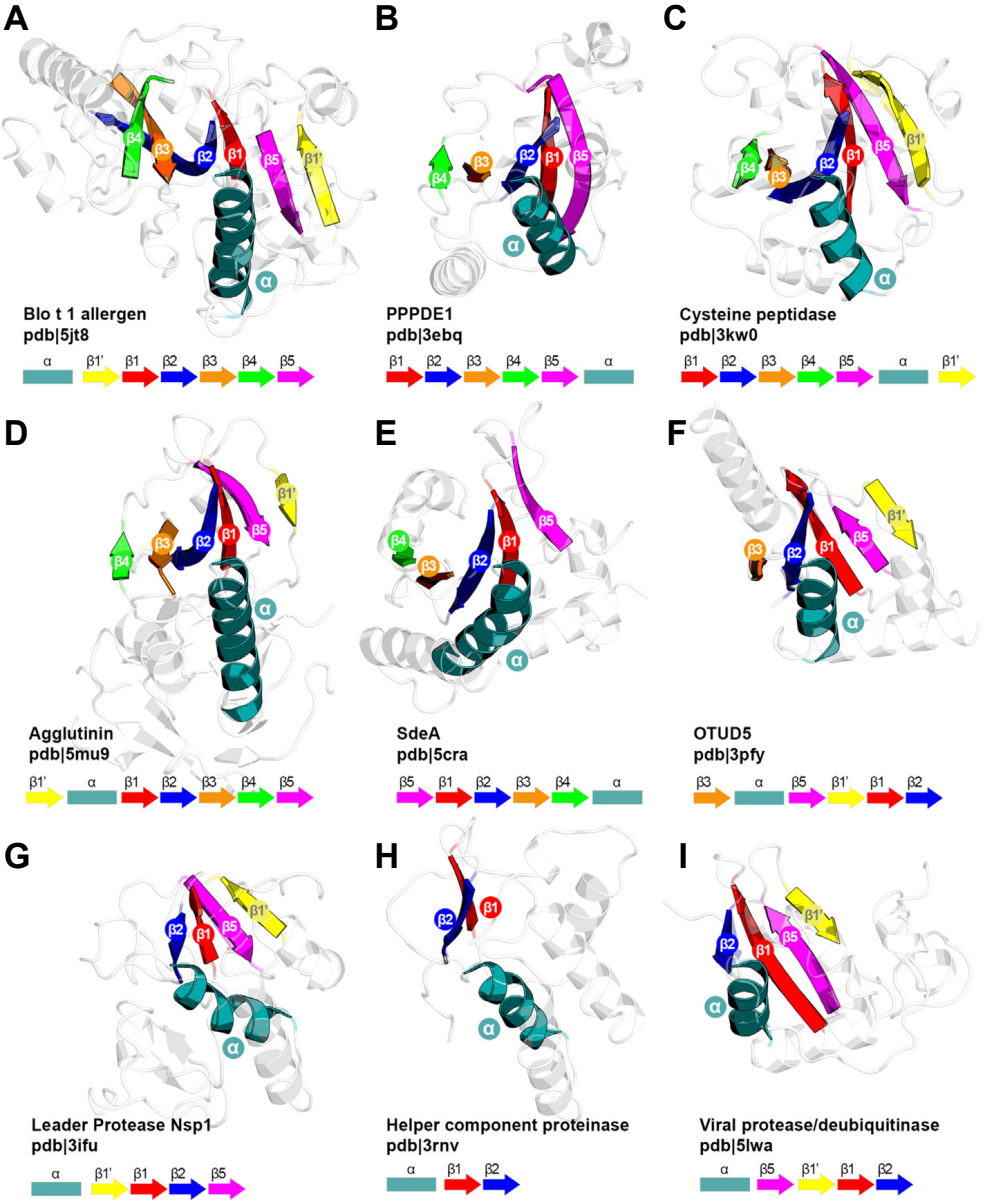
**Structural diversity of papain-like cysteine proteinases.** Corresponding core structural elements are rendered in the same colors. Under each 3D view, the order of core elements is provided to visualize multiple permutations observed within the superfamily. *A*, canonical papain-like structure of Blo t 1 allergen. *B*, circular permutation of α-helix in PPPDE1. *C*, circular permutation of α-helix along with β-strand 1' in cysteine peptidase from *Bacillus cereus*. *D*, β-strand 1' is additionally reversed in fungal agglutinin. *E*, *Legionella pneumophila* SdeA deubiquitinase with β-strands 4 and 5 reversed. *F*, human OTUD5 with β-strand 1' reversed, β-strand 3 and 5 permuted, the latter also reversed. *G*, reduced structure of viral protease Nsp1. *H*, even more reduced papain-like core from potyvirus proteinase retaining as little as α-helix and two β-strands. *I*, highly diverged protease from Turnip yellow mosaic virus missing β-strands 3 and 4, having β-strands 1' and 5 reversed, the latter additionally permuted.

Papain-like cysteine proteinases share a common structural core comprising α-helix followed by, depending on definition, four to five β-strands forming an antiparallel β-sheet. Based on structural alignments for the whole superfamily, we add an additional β-strand to this definition (β1'), which, despite being highly variable, is still present in the majority of the families (Fig. 3*A*, PDB ID: 5JT8). Quite unusually for protein structural classifications where fold definitions generally are clear and adequately represent common parts of protein structures, members of this superfamily adopt an extraordinary repertoire of alterations, which in many cases means lack of the alleged core elements. Commonly recognized are circular permutations of the α-helix alone or together with β1' (Fig. 3*B*, PDB ID: 3EBQ, and C, PDB ID: 3KW0) as had been thoroughly described for, for example, deubiquitinases (28) and NlpC/P60-like enzymes (29). The permutation events may also affect other elements that are often associated with the reversed direction of β-strands within the β-sheet. For instance, agglutinin from mushroom *Marasmius oreades* (34) (Fig. 3*D*, PDB ID: 5MU9) has β-strand 1′ permuted and reversed; *Legionella pneumophila* SdeA deubiquitinase (Fig. 3*E*, PDB ID: 5CRA (35)) lacks β-strand 1′, and its β-strands 4 and 5 have reversed direction, the latter being in addition permuted; human OTUD5 protein (Fig. 3*F*, PDB ID: 3PFY) β-strand 1′ is reversed, fourth β-strand missing, and β-strands 3 and 5 permuted—the latter in addition reversed. Interestingly, the third β-strand of OTUD5 still harbors active site glutamine.

Many viral papain-like peptidases are severely reduced in structure missing β-strands 3 and 4, for example, Nsp1 protease from porcine reproductive and respiratory syndrome virus (27) (Fig. 3*G*, PDB ID: 3IFU) or are minimized to contain only α-helix and two β-strands like potyvirus helper–component proteinase (Fig. 3*H*, PDB ID: 3RNV). Another viral peptidase/deubiquitinase from turnip yellow mosaic virus (36) (Fig. 3*I*, PDB ID: 5LWA) lacks β-strands 3 and 4, its β-strands 1′ and 5 are reversed, the latter being in addition permuted.

### Active-site variation

**Figure 4:**
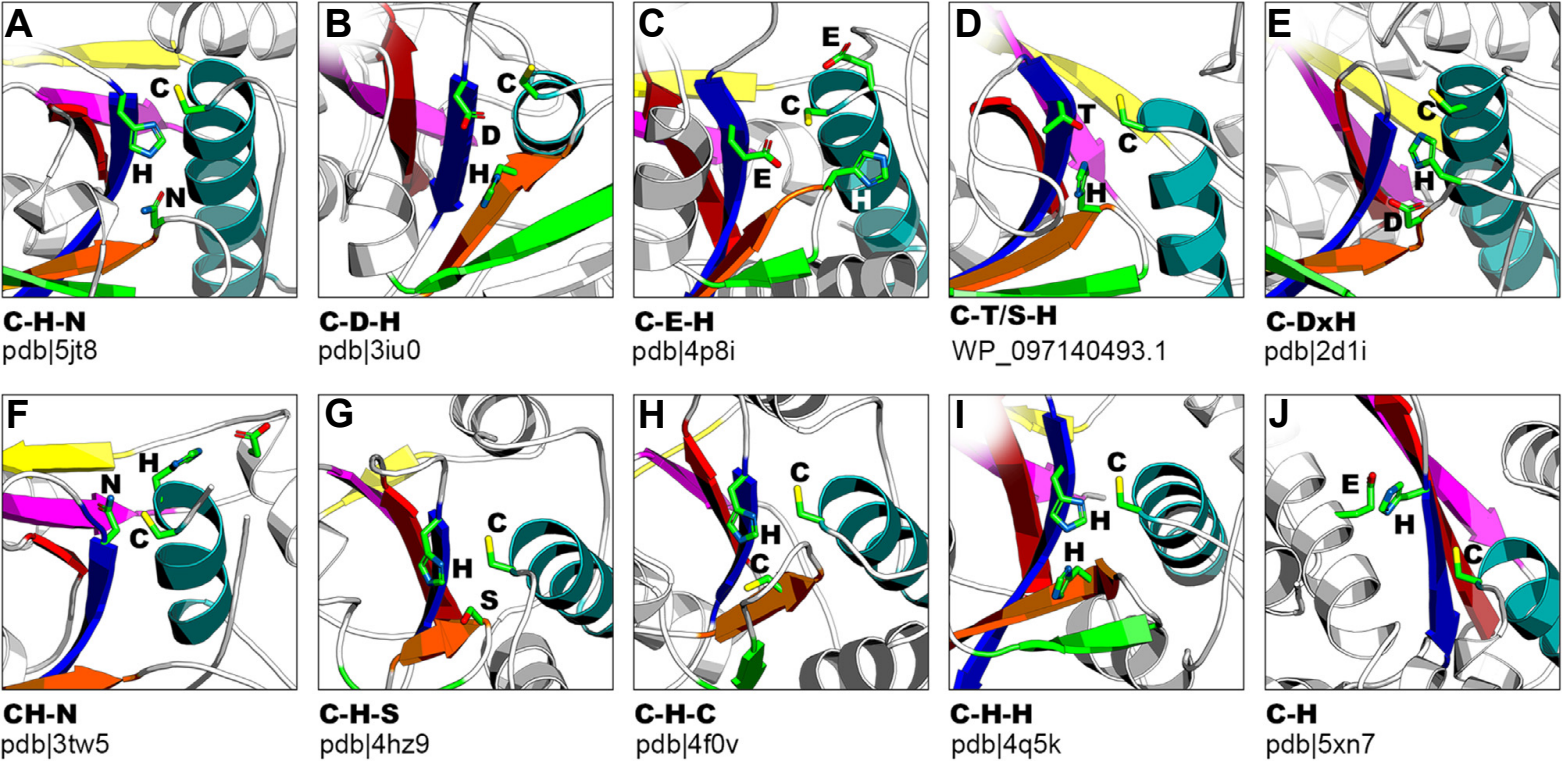
**Catalytic site variation.** Although the majority of papain-like peptidases retain canonical catalytic triad, multiple active-site migrations and structural permutations may be observed leading to a diversity of catalytic site architectures. *A*, canonical catalytic site in Blo t 1 allergen. *B*, microbial transglutaminase having histidine switched with aspartate. *C*, *Bacillus* transglutaminase may use one of two possible catalytic dyads. *D*, DUF1839 proteins retain hydroxyl group of serine/threonine in catalytic site. *E*, "Asp-Pro-His" box in human Atg4b. *F*, GP42 transglutaminase lacks histidine in its canonical position and now this residue is located right after cysteine to form the "CH" motif. *G*, Tea3 amidase effector has serine residue instead of bigger asparagine/glutamine in the third position of catalytic triad. *H*, Tae1 effector uses cysteine for stabilizing histidine's imidazole ring. *I*, DUF1460 amidase has two histidines in its active site. *J*, MARTX toxin lacks β-strand 3 and histidine is stabilized by glutamate located on the adjacent α-helix. Structural core elements are rendered in *colors*, and catalytic residues are shown in *sticks*. Under each figure, a catalytic signature is given; “x” and “-” stand for one and any number of residues, respectively.

The canonical catalytic triad of cysteine peptidase consists of cysteine, histidine, and third polar residue: asparagine, glutamine, aspartate, or glutamate, and is indeed present in the majority of papain-like families (Fig. 4*A*, PDB ID: 5JT8). Although cysteine residue is always located at the very N terminus of the core α-helix regardless of other structural aberrations, many alternative active-site architectures may be observed. The most obvious is the reduction to catalytic cysteine–histidine dyad characteristic of multiple peptidases associated with virulence, for example, C32, C31, C07, C42, C06, C42, C09, C23, C34, C36 as well as in OTU-like deubiquitinases (clusters V and VII). Many of these proteins lack papain-like structural core elements beyond the second β-strand (harboring histidine) or retained the following elements remnant or permuted. Nevertheless, the structural permutation of β3 does not impose the lack of a third catalytic residue as can be observed in C33, Ceg23, and LotA, neither does the presence of a complete structural core requires the triad, for example, C10, Amidase_6 (PF12671), C115, DUF2459 (PF09601), C97, and Calici_PP_N viral polyprotein (PF08405).

The catalytic histidine may migrate from its canonical position to nearby structural elements and even lose its catalytic function. The microbial transglutaminase from *Streptomyces* (Fig. 4*B*, PDB ID: 3IU0, PDB ID: 1IU4) has histidine residue switched with aspartate (forming CDH triad), and now, aspartate plays an essential role in catalysis (25). Similarly, transglutaminase from *Bacillus* (Fig. 4*C*, PDB ID: 4P8I) also uses glutamate instead of histidine; however, this protein may operate as well based on an alternative catalytic dyad of cysteine and another, directly preceding it glutamate (37). DUF1839 (Fig. 4*D*, PF08893) proteins retain C(T/S)H catalytic triad in which histidine switched its place with threonine or serine. According to 3D modeling, threonine/serine's hydroxyl group interacts with histidine and orients it against cysteine. In human Atg4b (Fig. 4*E*, PDB ID: 2D1I) (24) and UfSP1 (38) deconjugating enzymes, catalytic histidine is located on strand β3 after aspartate, resulting in CDH triad where aspartate and histidine form “Asp–Pro–His” box defining unique group of the papain-like superfamily. A highly uncommon catalytic site may be found in GP42 transglutaminase (Fig. 4*F*, PDB ID: 3TW5), which has its histidine moved away from the canonical location to the position right after cysteine to form the “CH” motif interacting with catalytically important asparagine (located on β2) or aspartate (located on other noncore α-helix) (23).

The third polar residue from the triad may also vary. In Tae3 amidase effector (Fig. 4*G*, PDB ID: 4HZ9 (39)), RavD effector deubiquitinase (PDB ID: 6NII (40)), and vasohibin (VASH; PF14822, PDB ID: 6JZC (41)), histidine interacts with the hydroxyl group of serine (CHS triad), which might also be the case for C27 (PF05407) and DUF4796 (PF16044) families. Tae1 amidase effector (Fig. 4*H*, PDB ID: 4F0V) in turn uses cysteine for stabilization of histidine's imidazole ring (42), whereas amidases DUF1460 (Fig. 4*I*, PDB ID: 4Q5K (43)), DUF1287 (PF06940), Amidase_5 (PF05382), NLPC_P60 (PF00877, PDB ID: 3NPF, PDB ID: 2K1G (44)), as well as DUF6540 (PF20174), LRAT (PF04970), and Ac81 (PF05820) employ histidine as the third residue (CHH triad). Eventually, the MARTX toxin lacks catalytic residue on β3 at all, and instead histidine might be stabilized by glutamate located on the adjacent α-helix (Fig. 4*J*, PDB ID: 5XN7).

**Figure 5:**
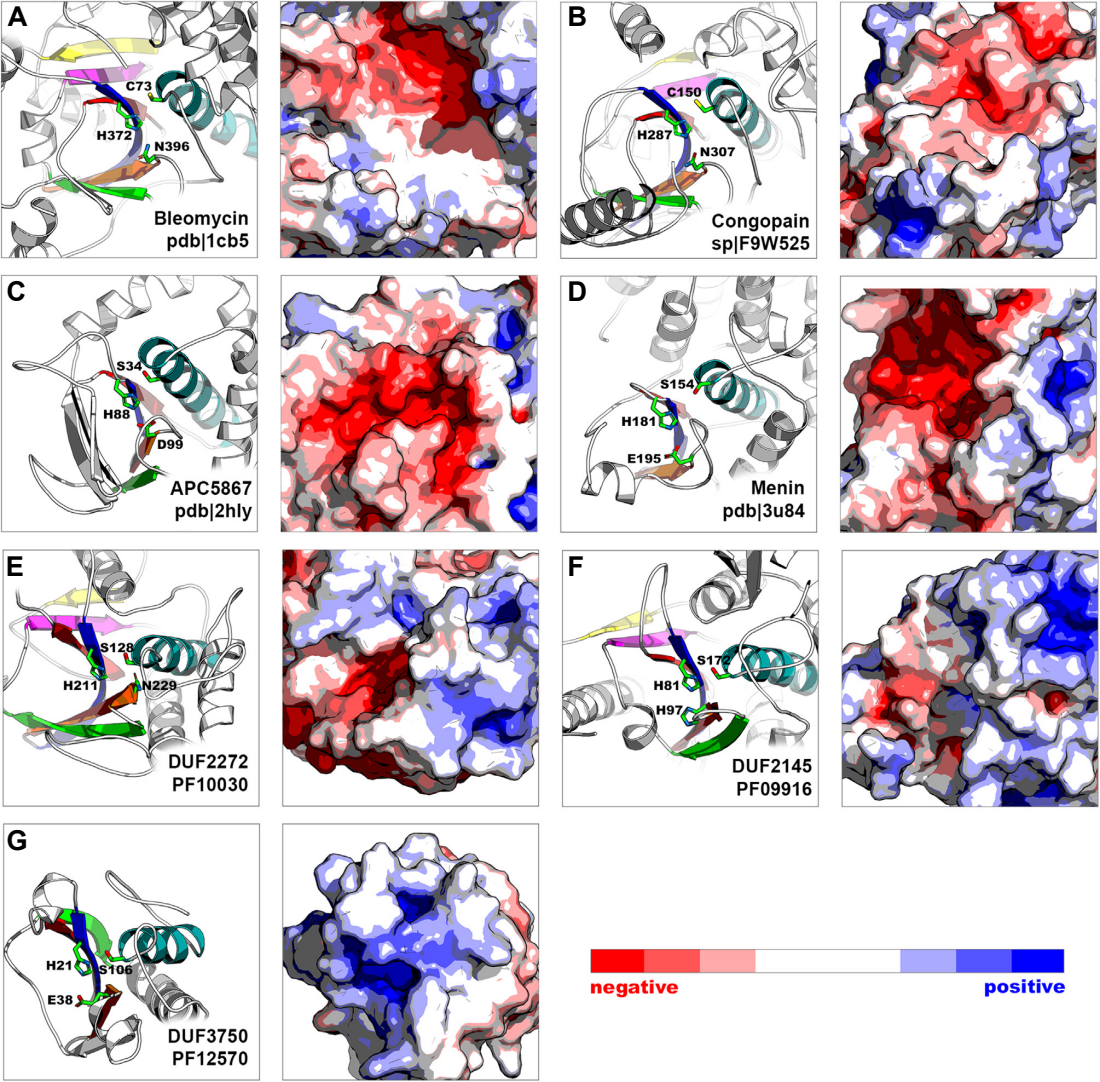
**Serine-based papain-like representatives.***A*, bleomycin and (*B*) congopain have cysteine residue in their catalytic triads but retain partial enzymatic activity after cysteine is mutated to serine. Both enzymes have negatively charged active-site pockets. Serine-based papain-like proteins: (*C*) APC5876, (*D*) menin, and (*E*) DUF2272 are also negatively charged around serine and hence might still function as peptidases. On the contrary, (*F*) DUF2145 and (*G*) DUF3750 are rather positively charged.

Several papain-like families: menin (PF05053, PDB ID: 3U84 (45)), structural genomics protein APC5867 (PDB ID: 2HLY), DUF2145 (PF09916), DUF2272 (PF10030), and DUF3750 (PF12570) have conserved serine residue in place of cysteine next to the two remaining active-site residues. Experiments for human bleomycin hydrolase and *Trypanosoma* congopain demonstrated that despite C->S mutation, these enzymes displayed non-negligible activity (46, 47). According to the results of Gisdon *et al.* (48), papain-like cysteine peptidase might retain its catalytic function with C->S substitution if the active site is more negatively charged as observed in serine peptidases. Both congopain (SP: F9W525) and bleomycin hydrolase (PDB ID: 1CB5 (49)) are negatively charged within the active-site pocket (Fig. 5, *A* and *B*). Similarly, APC5867 protein from *Agrobacterium fabrum* (Fig. 5*C*, PDB ID: 2HLY, PF09641), human menin (Fig. 5*D*, PDB ID: 3U84 (45), PF05053), and hypothetical protein B7Z80_21685 from *Rhodospirillales* bacterium (Fig. 5*E*, DUF2272, PF10030) display extensive patches of negative charge within active-site pocket suggesting that these proteins might function also as peptidases or peptidase-like enzymes. DUF2145 domain–containing protein from *Giesbergeria anulus* (Fig. 5*F*, PF09916) and DUF3750 domain–containing protein from *Rhodospirillales* bacterium (Fig. 5*G*, PF12570) are more positively charged and possibly would require specific substrates for reshaping potential active-site properties for catalysis. Nevertheless, these hypotheses remain speculative, and experimental research is required to provide the final proof.

### Major groups of papain-like proteins

#### Papain, cathepsins, calpains, staphopain, and others

Cluster I embraces papain itself, cathepsins involved in protein turnover, tissue remodeling, and homeostasis (50, 51, 52), and calpains—Ca-dependent peptidases important for remodeling tissues, cell cycle progression, cell mobility, and proteasome-independent protein degradation (53, 54). It also includes enzymes that deconjugate proteins from lipids (24, 55), peptides during translocation (56, 57), or ubiquitin-like proteins (38, 58, 59). This cluster contains multiple virulence and defense factors like staphopain A cleaving, for example, host elastins found in connective tissues and pulmonary surfactant protein A in the lungs (60, 61), effector from *Erwinia amylovora* critical for plant–pathogen interactions (62), butirosin biosynthesis protein H protecting antibiotic butirosin from several common resistance mechanisms (6, 63), or bacteriophage-encoded virulence factor GtgE disrupting the development of lysosome-related organelles required for delivering antimicrobial factors (64). Proteins from cluster I retain all core structural elements, with additional conserved α-helices inserted between the core α-helix and β1' and between β1' and β1. An extended β-hairpin between β1 and β2 and the presence of well-pronounced β4 allow the β-sheet to fold into a partially open barrel-like structure.

The C1 peptidases (PF00112, PF03051) include two closely related protein families, PF00112 (C1_1) and PF03051 (C1_2). C1_1 contains papain-like endopeptidases and cathepsins, whereas C1_2 groups aminopeptidases like bleomycin hydrolase (65) and pepW (66). Calpains (C02, PF00648) are calcium-dependent nonlysosomal cysteine proteases (67). Although the physiological purpose of calpains is still unknown, it has been demonstrated that they are active players in cell mobility, cell cycle progression, long-term potentiation in neurons, and cell fusion in myoblasts; calpains get activated by a localized influx of calcium into the cell, which then progresses the signal transduction pathway by catalyzing the regulated proteolysis of their target proteins (68). C54 (PF03416) and C78 (PF07910) proteins are involved in maturation and subsequent recycling by deconjugation of ubiquitin-like proteins or lipids (24, 38, 55, 58, 59). C66 (Mac-1, PF09028) contains bacterial proteins, for example, IdeS—an endopeptidase specifically degrading immunoglobulin G, which blocks opsonophagocytosis (69). C10 (PF01640) peptidases are bacterial virulence factors degrading immune effector proteins and destroying tissues, for example, interpain A (70) or SpeB (71). C102 family, represented by GtgE protease from *S. typhimurium* (PDB ID: 5OED_A)—a type II secretion system effector protein cleaving Rab32 involved in the development of lysosome-related organelles, which prevents the delivery of antimicrobial factors (64).

Also, a subcluster of closely related protein families may be defined. C47 (PF05543) family contains staphopain A, which cleaves host elastins found in connective tissues, pulmonary surfactant protein A in the lungs, and the chemokine receptor CXCR2 on leukocytes. Proteolytic cleavage of surfactant protein A impairs bacterial phagocytosis by neutrophils, whereas CXCR2 degradation blocks neutrophil activation and chemotaxis (60, 61). C70 (PF12385) proteins are bacterial peptidases important for plant–pathogen/pest interactions, for example, type III effector protein from *E. amylovora* attacking *Malus robusta* (62). Guanylate cyclases (Guanylate_cyc_2, PF09778) catalyze the conversion of GTP to cGMP, for example, Arabidopsis GC1 protein (72). Human ortholog, GUCD1 (LLN4), interacts with NEDD4-1 affecting tumor suppressors (73) and lacks the domain providing the cyclase function described for GC1. Hence, the biological function of this papain-like domain within this family might not be connected to cyclase explicitly. The DUF3335 (PF11814) family is closely related to PF09778 discussed previously. Its members are often fused to the GCN5-related *N*-acetyltransferase family domains, which might function as peptide ligases or acetylates (74). C39 peptidases (PF03412) are fused to ABC transporters removing double-glycine type leaders from secreted proteins during translocation (56). These family members have reduced structure compared with others from cluster I, which might be an accommodation to functioning as a part of the transporter (75). In contrast, the closely related family, C39_2 (PF13529), contains single-domain peptidases with more structural decorations, probably for substrate specificity. Nevertheless, it still is associated with transporters—for instance, it can be located close to genes encoding teichoic acid ABC transporter permease, YxeA (COG5294, PF06486, homolog of membrane-associated BcR97A protein; PDB ID: 2K5Q), nitroreductase, MFS transporter, and PTS sugar transporter subunits. Bacterial ABC and peptide MFS transporters are used, for example, for acquiring nitrogen and amino acids (57). BrtH_N (PF14399) proteins are closely related to the C39 and C70 families. The BtrH_N domain is part of the acyl carrier protein:aminoglycoside acyltransferase BtrH, which protects the antibiotic butirosin from several common resistance mechanisms (6, 63). Phytochelatin synthase (C83, PF05023) catalyzes the deglycination of a GSH donor molecule in the process of synthesis of heavy-metal–binding peptides (phytochelatins) from glutathione and related thiols (76). This domain is often fused to Phytochelatin_C (PF09328) of unknown function.

#### NlpC/P60, CHAP, and amidases

This cluster groups enzymes that process proteins attached to peptidoglycans. They facilitate bacterial cell division, modulate microbe–host interactions, act as virulence factors (39, 77, 78), degrade peptidoglycans (79, 80), remodel cell wall (81, 82), deSUMOylate (83, 84), or disassemble plant cell wall in herbivore's rumen (85).

Cluster II proteins retain characteristic “GD” sequence motifs localized to β-strand 1. The aspartate interacts with arginine on β-strand 5, which might stabilize the structure. These proteins have shorter β-strands 3 and 4 compared with cluster I and form a more compact domain. Interestingly, this cluster splits into two major structural groups—canonical and circularly permuted (with permuted α-helix and the first β-strand). Despite the permutation, both groups have biological functions generally related to the cell membrane.

NlpC/P60, CHAP, and several other families are a group of closely related proteins within cluster II. NlpC/P60 proteins (C40, PF00877) are cell-wall–associated dipeptidyl endopeptidases cleaving peptidoglycans (30). *Mycobacterium tuberculosis* uses NlpC/P60 protein for cell wall remodeling, bacterial daughter–cell separation, as well as for degradation of *Bacillus* peptidoglycan (81); mammalian commensal *Enterococcus faecium* uses it also for cell wall remodeling, which alters immune response in mammalian intestinal cells (82). They are present also in nonbacterial species, for example, in *Trichomonas vaginalis*, which upregulates in the presence of bacteria (86). CHAP family (C51, C104, and PF05257) groups multiple amidases and peptidoglycan hydrolases (87). Cell wall–degrading enzymes include *Enterococcus faecalis* phage endolysin (PDB ID: 6IST), which specifically cleaves the host's cell wall in order to release phage progeny during the lytic cycle (77, 78), lysin from staphylococcal phage GH15 (PDB ID: 4OLK) lytic against methicillin-resistant *Staphylococcus aureus* (88), phage cell-wall specific lysin PlyC (PDB ID: 4F88) (89), PcsB—a streptococcal peptidoglycan hydrolase splitting septal cross wall in cell division and daughter cell separation (PDB ID: 4CGK) (90). Other functions of CHAP proteins include glutathionylspermidine synthetase (*E. coli*, PDB ID: 2IO7) (91) and trypanothione biosynthesis–related amidase (*Leishmania major*, PDB ID: 2VPM) (92). According to sequence similarities, representatives of C51 MEROPS family are closely related to phage lysins (PDB IDs: 6IST and 4OLK), C104 to lysin PlyC (PDB ID: 4F88), whereas C51.A to glutathionylspermidine synthetase (PDB ID: 2IO7), and trypanothione synthetase (PDB ID: 2VPM). Another peptidoglycan-related domain, bacteriophage peptidoglycan hydrolase (Amidase_5, PF05382), found mainly in Caudovirales and Firmicutes is often fused to immunoglobulin-like ZoocinA_TRD (PF16775) important for cell wall recognition upon bacterial invasion (79). Amidase_6 proteins (PF12671) present in Firmicutes and Actinobacteria function as amidase effectors, for example, Tae3 (PDB ID: 4HZ9), which cleaves peptidoglycan to disrupt bacterial cell wall upon infection (39). DUF2272 (PF10030) representatives, mostly proteobacterial, may degrade plant cell wall for bacteria living in herbivores' rumen (PDB ID: 4EYZ) (85) or function as peptidoglycan amidase secreted to the periplasmic space of a competitor (Tse1, PDB ID: 4F0V) (42, 93). Eventually, this group also includes two families of unknown function: inactive DUF1175 (PF06672) binding to peptidoglycan (94) and bacterial family of unknown function DUF1287 (PF06940).

Protein–glutamine gamma-glutamyltransferase (TGL, PF20085) catalyzes protein cross-linking reactions that modify chemical and physical properties of cellular structures, for example, bacterial spore coat transglutaminase Tgl (PDB ID: 4P8I), which ligates glutamine to lysine by forming an amide bond (37).

Homologs of type 6 secretion amidase effector 2 (*Salmonella enterica*, Tae2, PDB ID: 6WIN) form a new family of bacterial and eukaryotic proteins. Tae2-like effector was domesticated, for example, by *Ixodes scapularis*, black-legged tick, which uses it to selectively kill mammalian skin microbes (95).

Circularly permuted representatives of cluster II belong mainly to C97, C92, and LRAT families. C97 proteins (PPPDE putative peptidase domain, DUF862, PF05903) function as deubiquitinases/desumolyases, for example, murine Desi-1 (PDB ID: 2WP7 (84))—deubiquitinase that also deconjugates SUMO1, SUMO2, and SUMO3 from its substrate proteins (83) and plays role in UBIN/POST system responsible for exporting of polyubiquitinated proteins from the nucleus (96). C92 peptidases (PF05708) are lipid amidases. A representative from *Bacillus cereus* (PDB ID: 3KW0) cleaves the amide bond between a lipid and an amino acid (29). LRATs (PF04970) function as lipid-metabolizing enzymes and cleave at the lipid membrane–water interface. The human genome encodes six LRAT-like homologs involved in, for example, lipolysis regulation (97), vitamin A processing (97), or tumor suppression (98). Remaining cluster II families include viral polyprotein N-terminal (Calici_PP_N, PF08405), a small family of viral accessory proteins in the N terminus of ORF1 polyprotein, as well as multiple newly annotated families, described in further sections.

#### Acetyltransferases

Cluster III groups acetyltransferases, mostly deamidases/transglutaminases, functioning as toxic virulence factors (2, 13, 14, 99, 100, 101, 102). They are also involved in post-translational protein modifications (41), protein degradation, angiogenesis (103, 104), and blood clotting (105). Catalytically inactive families take part in transcription regulation networks or scaffold nucleotide excision repair complexes (106, 107).

Proteins from cluster III display structural diversity resembling diverse biological functions and targets. For instance, detyrosinase Vash2 has a number of structural decorations, which bury active-site pocket within deep and positively charged well specific for negatively charged tubulin (PDB ID: 6JZC (41)), whereas *Pasteurella multocida* toxin is smaller, with the catalytic site more freely available from multiple directions and surrounded by patchy electrostatics (PDB ID: 2EBF (13)). The majority of cluster III families lack β-strand 1′ as well as canonical residue stabilizing oxyanion hole.

Multiple transglutaminase families have been described as bacterial toxins. Papain fold toxin 2 (Tox-PL-2, PF15643) is identified in diverse secretory systems of parasitic bacteria, where they appear to be directed toward host cells, in addition to toxins from free-living bacteria. These enzymes may either catalyze a traditional thiol peptidase reaction or operate as transglutaminases, mediating protein crosslinking *via* a transglutaminase reaction (14). Representative of the family Tox-PLDMTX (PF15645), SseI from *Salmonella* is secreted into host cells and contributes to the establishment of systemic infections and probably acts as Gln deamidase (99). DUF2026 (PF09641) is a family of unknown function; however, its genomic neighborhood in multiple bacterial species (restriction endonuclease, misannotated as SMEK domain–containing protein; hypothetical protein, *e.g.*, WP_016263567.1 containing PIWI-like domain; DUF4354 from MPT63-MPB63 Pfam clan, which includes also secreted and immunoglobulin-like MPT63 protein from *M. tuberculosis* involved in virulence (108)) suggests that DUF2026 proteins might function as peptidase-like virulence factors. C116 peptidase, *P. multocida* toxin (PDB ID: 2EBF), is also a virulence factor exerting its toxicity in a yet unknown way by upregulating different signaling cascades downstream of the heterotrimeric GTPases Gq and G12/13 (13). Gln_deamidase_2 (PF18626, PDB ID: 3A54) are protein glutaminases (109) with a deficiency in organelle trafficking/intracellular multiplication of type IVb secretion system in their genomic neighborhood. Deficiency in organelle trafficking/intracellular multiplication is an essential virulence factor for the intracellular lifestyle of bacteria used for translocating over 300 effector proteins into host cells (110). Gln_amidase (PF15644) representatives are also observed in bacterial toxin systems where they might act as a releasing peptidase or a poison (14, 111). OspI (PDB ID: 3B21) is a bacterial glutamine deamidase that selectively deamidates UBC13 protein and eventually dampens acute inflammatory response (111, 112).

The following six closely related protein families form a subgroup within cluster III. C93 peptidases (PF06035) catalyze post-translational protein modification *via* transamidase, acetylase, or hydrolase activity (113): C-di-GMP (PDB ID: 4FGP) is a key molecule in controlling bacterial motility and biofilm formation (114). Transglut_core (PF01841) family contains, for example, transglutaminase important for virulence of *Streptococcus* (Ss2, PDB ID: 4XZ7) (101), tgpA from *Pseudomonas aeruginosa* attached to the inner membrane and important for bacterial survival (PDB ID: 6G49) (101), human TGase activated by Ca^2+^ ions (PDB ID: 1L9M) (115), coagulation factor XIII (PDB ID: 4KTY) targeting enzymes in the blood-clotting cascade, factor Xa and thrombin (105). PF01841 covers at least three MEROPS families (C110, C111, and C113), which might reflect multiple expansions of TGases in many species. PeiW and PeiP from *Methanothermobacter wolfeii* belong to the C71 family (PF12386) and catalyze the lysis of *Methanothermobacter marburgensis* cells—they cleave the ε-isopeptide bond between alanine and lysine in pseudomurein (100). Representatives of this family are also found in viruses and are probably important for releasing virions after infection (2). EDR1 (PF14381) proteins are highly similar to C71 and C93 peptidases and are often fused to the kinase domain (PK_Tyr_Ser-Thr, PF07714). EDR1 has previously been identified as a negative regulator of disease resistance and ethylene signaling (116). DUF553 (PF04473) proteins catalyze post-translational modifications of proteins by transferring, for example, peptidyl glutamine residues to a variety of amines (117). C96 family (Transglut_core3, PF13471) includes *Escherichia coli* McjB, which proteolytically processes McjA into a mature antimicrobial factor (118).

VASHs (PF14822) are eukaryotic proteins discovered as calcium-dependent secreted angiogenesis regulators (103, 104, 119). VASH2 (PDB ID: 6JZC) removes tyrosine from tubulin, prolonging microtubule lifetime by protecting microtubule from depolymerizing motor proteins (41). Another solely eukaryotic family, menin (PF05053), is involved in the regulation of transcriptional networks. Menin protein is an essential component of an MLL–SET1 histone methyltransferase complex, binds to the TERT promoter, and represses telomerase expression, plays a role in transforming growth factor B1–mediated inhibition of cell proliferation, represses JUND-mediated transcriptional activation, and regulates HOXC8 and HOXC6 gene expression (120). Deficiency of menin results in multiple endocrine neoplasia 1 (45), but on the other hand, its inhibition effectively disrupts leukemogenic transcriptional networks, resulting in the synergistic killing of leukemia cells (121). Human menin lacks catalytic cysteine residue and probably is catalytically inactive, but some other family members retain full catalytic triad. Menin is known as a scaffolding protein in cell signaling (122).

Acetyltransf_2 (PF00797) proteins participate in the detoxification of a plethora of hydrazine and arylamine drugs; they catalyze the N- or O-acetylation of various arylamine and heterocyclic amine substrates. Arylamine *N*-acetyltransferase from *Mycobacterium abscessus* (PDB ID: 4GUZ) is an intracellular xenobiotic-metabolizing enzyme for the acetylation of aromatic amines. In humans, these enzymes take part in drug metabolism where they acetylate and inactivate various antibacterial substances, including antituberculosis drugs (123).

The function of the Transglut_core_2 (PF13369) family remains unknown. Human FBXO21 protein mediates the ubiquitylation and proteasomal degradation of EID1 (124), but the detailed roles of bacterial family representatives are unknown.

The Rad4 (PF03835) family is closely related to PF01841. DNA repair protein RAD33 (PDB ID: 7K04) has an inactive papain-like domain for DNA binding within the TFIIH/Rad4–Rad23–Rad33 complex in nucleotide excision repair (106, 107). However, another family member, yeast PNGase (PDB ID: 3ESW), is a peptide:N-glycanase, which deglycosylates misfolded glycoproteins within the endoplasmic reticulum–associated protein degradation pathway (125).

Nt_Gln_amidase (PF09764, PDB ID: 4W79) mediates the side-chain deamidation of N-terminal glutamine residues, which is an important step in the N-end rule pathway of protein degradation (126).

#### Deubiquitinases

Cluster IV groups multiple deubiquitinating enzymes. Closely related are families of eukaryotic proteins UCH (PF00443) and UCH_1 (PF13423), whereas Peptidase_C98 (PF15499) remains more remote to the two. All the proteins have an extensive insertion between the core α-helix and β-sheet—it contains a β-sheet forming a “finger” domain whose major role is to grip the ubiquitin. Structurally, they have a rather straight β-sheet—they have a bigger insertion between β1' and β1, which prevents the formation of a more curved barrel-like structure; this insertion also seems to stabilize the “fingers.” UCH_1 family members often have additional RnaseT domain, whereas Peptidase_C98 representatives specifically are fused to the DUF4650 (PF15509).

Deubiquitinating enzymes underwent significant expansions and occur in multiple copies per genome. For instance, humans have tens of DUBs (15, 16) divided into multiple classes: OTU2 (peptidase C65, PF10275), USP (UCH, PF00443), UCH (peptidase C12, PF01088), MJD (Josephin, PF02099), and JAMM (Prok-JAB, PF14464 belonging to metalloproteases). Despite the overall similarity in structure and catalytic sites, they are specialized, for example, USP1 removes monoubiquitin from FANCI-FANCD2 during DNA repair processes (127), USP8 hydrolyzes monoubiquitylated and polyubiquitylated epidermal growth factor receptors (26), USP15 takes part in transforming growth factor-beta signaling, mitophagy, mRNA processing, and innate immune responses (128), and USP30 is a negative regulator of mitophagy as it antagonizes Parkin-mediated ubiquitination events in damaged mitochondria (129). Some family members are involved in pathways related to nucleic acid processing like UBP8, which is part of SAGA transcriptional coactivator complex where it deubiquitinases histone H2B (130). They also function as now inactive domains providing structural scaffolding, for example, Sad1 positioning Brr2 helicase within U4/U6.U5 tri–snRNP complex (131) or PAN2 fused to RnaseT domain in deadenylating complex (132).

Along with specialization come specific regulatory mechanisms. USP14 becomes activated when interacting with 26S proteasome where it rescues mistakenly ubiquitinated proteins from degradation (133). USP25 has a long coiled coil between β1′ and β1, which promotes tetramerization as an autoinhibitory mechanism (134). USP35 has two isoforms that localize to different intracellular compartments and have distinct functions, one being an antiapoptotic factor and the other regulating lipid homeostasis (135).

A leader protease of the foot-and-mouth disease virus (C28, PF05408, PDB ID: 1QUL) targets the host's eIF4G. It prevents the host's native translation but does not alter viral translation, which relies on IRES sequence and its interaction with the C-terminal region of eIF4G domain (136). Its structure is quite minimal, with an accessible active site without any bigger loops.

CoV_peptidase (PF08715) and Peptidase_C16 (PF01831) are accessory peptidases processing the replicase polyprotein in coronaviruses, for example, papain-like peptidases from SARS Replicase polyprotein 1ab (PDB ID: 2FE8), which is structurally very similar to other deubiquitinating enzymes and retained the “fingers” domain (137). It has deubiquitinating activity (138), so it might affect the host's ubiquitination machinery during the infection. CoV_peptidase is more abundant, and in some viruses, it co-occurs with another peptidase, C16 (139).

#### Deubiquitinases, deamidases

Cluster V includes multiple viral peptidases: C34 (PF05413) from plant-infecting closteoviruses (5), C33 (PF05412) polyprotein peptidases involved also in deubiquitination of host proteins (10, 140), C23 (PF05379) carlavirus (apple stem pitting virus) peptidase (141), DUF1717 (PF05414) from apple stem grooving virus also known as citrus tatter leaf virus, C21 (PF05381) endopeptidases from turnip yellow mosaic virus–related viruses, which in addition to polyprotein processing have deubiquitinating activity (PDB ID: 5LWA) (36), C36 (PF05415) peptidase of beet necrotic yellow vein furovirus (142). It also covers the OTU-like cysteine protease family (PF02338) containing three MEROPS families: C64, C85, and C87. C64 peptidases are eukaryotic endoisopeptidases that release ubiquitin from ubiquitinated proteins in multiple signaling pathways (7, 143, 144), and C87 peptidases are viral deubiquitinases (*e.g.*, Crimean Congo hemorrhagic fever virus, PDB ID: 3PHU), which disrupt ubiquitin-dependent antiviral signaling pathways (145). C65 family (PF10275) consists of eukaryotic deubiquitinases, for example, OTUB1 that regulates the synthesis of K63-linked polyubiquitin chains at double-strand break sites (146) and OTUB2 processing a wide spectrum of ubiquitin moieties (147). C101 (PF16218) proteins function also as deubiquitinases, for example, human OTULIN/Gumby/Fam105B (PDB ID: 4KSK), which alters NF-κB-dependent transcription and affects angiogenesis (148). The inactive paralog of OTULIN–FAM105A/OTULINL (PDB ID: 6DRM) lacks DUB function and localizes to endoplasmic reticulum and nuclear envelope (149). C119 includes *L. pneumophila* lpg2249/Lem27 protein, which alters the host ubiquitin network to form the *Legionella*-containing vacuole for reproduction (PDB ID: 7BU0) (150) and lpg2248/LotA bacterial effector protein with two OTU domains having preferences for different ubiquitin moieties (PDB ID: 7F9X) (151).

One family from cluster V contains circularly permuted proteins—LupA (PF18242) named after *Legionella* ubiquitin-specific protease A, which deubiquitinates its cognate effector LegC3 and affects vesicle trafficking (152). LupA is closely related to LotA, and despite being a circular permutation, it displays high structural similarity.

Cluster VI contains C55 (PF03421) and C122 (PF19049) bacterial effectors. C55 proteins function as acetyltransferases, for example, *Salmonella* AvrA suppressing JNK signaling (153), and *Yersinia* YopJ—a serine/threonine acetyltransferase disrupting innate immune response (154). C122 enzymes are deubiquitinases, for example, *L. pneumophila* SidE interfering with the host's ubiquitin network upon infection (35).

Cluster VI includes also a group of closely related families: C05 (adenain, PF00770) cleaving viral precursor proteins and involved in uncoating and targeting the host's cytoskeletal keratins (155, 156), C120 (mavirus peptidase), C57 (PF03290) Poxviridae core proteinase (157), C63 (PF02902) African swine fever virus pS273R protease processing polyprotein precursors (158), C79 (PF02902) bacterial effectors (*e.g.*, *E. coli* protein ElaD and *Salmonella* SseL) (159) as well as Ulp1 protease family (C48, PF02902)—eukaryotic proteases specific for removing ubiquitin and ubiquitin-like modifiers like SUMOs and NEDD8 from their targets, for example, sentrin-specific proteases (SENP6 and SENP7) cleave SUMO2 and SUMO3 (160), Den1 selectively cleaves NEDD8 (161), Ulp1 cleaves SUMO (162), and DUB1 from *Chlamydia* deconjugates ubiquitin and NEDD8 (159) but also functions as Lys-acetyltransferase (163).

Clusters VII and VIII group viral accessory peptidases. C42 peptidase (PF05533) from *Closteroviridae*, for example, L-pro from beet yellow virus, despite its autocatalytic function, is also involved in systemic transport and RNA amplification (164). In some viruses, L-pro is duplicated, and both peptidases now become specialized—one directly taking part in infection, and the other playing accessory roles (165). C06 (PF00851) are accessory peptidases in *Potyviridae*, for example, HC-Pro from bean common mosaic necrosis virus. C31 (PF05410), porcine reproductive and respiratory syndrome arterivirus–type cysteine peptidase alpha (lactate dehydrogenase–elevating virus), inhibits host interferon-beta production by degrading CREBBP transcriptional activator, participates in the inhibition of host NF-κB activation by counteracting LUBAC-dependent induction of NF-κB, and reduces host NEMO ubiquitination by blocking the interaction between the two LUBAC complex components RNF31 and SHARPIN (166, 167). It also is important for blocking host's mRNA synthesis (168). C32 (PF05411) Nsp1-beta equine arteritis virus–type cysteine peptidase is one of the two accessory proteases encoded in the PRRSV genome, important for viral RNA synthesis (168) and inhibiting interferon-activated Janus kinase/signal transducer and activator of transcription protein signal transduction by mediating the ubiquitination and subsequent proteasomal degradation of host KPNA1 (169). It has an N-terminal and Mn-dependent nuclease domain, whereas a C-terminal peptidase domain is required for self-release (170).

Clusters IX and X include families of bacterial effectors. C58 (PF03543) are avirulence proteins required for the infection of plant cells (171, 172, 173). C118 (Toxin_15, PF07906) representative, *Shigella* T3SS effector OspD3, cleaves RIPK1 and RIPK3 kinases to prevent necroptosis (11). PatoxP (PDB ID: 6HV6) is an effector from the entomopathogenic bacterium *Photorhabdus asymbiotica* having a toxic effect on animal cells (174). Cycle inhibitory factors (PF16374) are bacterial effectors causing an irreversible cell cycle halt by inhibiting the degradation of the cyclin-dependent kinase inhibitors p21 and p27. They are protein–glutamine deamidases targeting ubiquitin or the ubiquitin-like protein NEDD8 regulating CLR complexes (175). Deamination of NEDD8 makes it immune to degradation, and in consequence, leads to cell cycle arrest (176). MavC from *L. pneumophila* (PDB ID: 7BXF) is a bacterial effector functioning as ubiquitin deamidase and noncanonical ubiquitinase and deubiquitinase of UBE2N protein important for inflammatory and DNA damage response pathways (177).

### Newly identified proteins and families

We found 21 yet unclassified protein families to retain papain-like domain (for more details on each family, see supporting information). These include nine bacterial DUFs lacking any experimental data on their potential biological importance. According to genomic contexts, DUF6005 might be related to siderophore biosynthesis, DUF1839 might function at a cell wall processing glycans in acyl-CoA-dependent manner, DUF2459 might be part of urease-related pathway, and DUF4105 might support resistance transporters or translocation process. DUF4846 in turn might be another NlpC/p60-like cell wall–degrading/modifying domain as it retains the “GD” motif characteristic for this class of enzymes. In addition, four viral families contain presumably accessory proteases important for virulence and development.

Among newly identified families, seven are eukaryotic, containing also uncharacterized human proteins. For instance, C14orf28 (dopamine receptor–interacting protein, DRIP1 (178)) retains papain-like deubiquitinase domain with additional, second “fingers”-like domain. DUF7788, a family of uncharacterized transmembrane proteins, includes not only *A. thaliana* RTE1 (ethylene signaling (179, 180, 181)) but also human TMEM222 (C1orf160) predominantly found in brain (182), and deleterious variants of which correlate with neurodevelopmental disorders (183). Human CEP76 and DRC7 from KOG3639 family contain potentially active papain-like domain and might be involved in altering centriole dynamics. Their inactive homolog, CC2D2A, is critical for cilia formation and might be involved in Ca^2+^-mediated signaling (184).

### Human enzymes

The human genome encodes 192 papain-like proteins. The most abundant are enzymes processing/deconjugating ubiquitin or ubiquitin-like modifiers: 20 U17-like and 49 UBP-like ubiquitin hydrolases, C14orf28 of unknown function, CYLD signaling protein (185) (cluster IV), 16 OTU-like deubiquitinases involved in signaling and glycosylation (and two inactive homologs: vertnin and OTULL) (186, 187) (cluster V), seven SENPs processing and deconjugating ubiquitin-like proteins (SUMO or NEDD8) in multiple biological contexts (188) (cluster VI), as well as several DUB families not belonging to any cluster: BAP1 histone H2A deubiquitinase (189), UCH-like proteins (UCHL5-associated proteasome regulation (190), UCHL1 (191) and UCHL3 cleaving ubiquitin or NEDD8 (192)), MINDY hydrolases (MINY4, MINY1 (193), and MINY2 (193)), and four Josephin-related proteins (194).

DUBs may be also found in cluster I: ZUP1 is an endodeubiquitinase taking part in maintaining genome stability and cellular survival in response to exogenous DNA damage (195). Cluster I groups also 14 calpains and 11 cathepsins, four ATG4 proteins involved in autophagy by mediating both proteolytic activation and delipidation of ATG8 (196), BLMH bleomycin hydrolase (65), BIVM (197, 198), GUCD1 (LLN4) interacting with NEDD4-1 (73), C19orf54 of unknown function, and UFSP2, which hydrolyzes UFM1, a ubiquitin-like protein (199). Cluster I contains also several inactive papain-like proteins: androglobin, two tubulointerstitial nephritis antigen proteins (TINAG, TINAL) affecting proximal tubule epithelial cell interaction with surrounding matrix (200), and UFSP1—a homolog of UFSP2 mentioned previously.

Cluster II of NlpC/P60-like amidases includes MKROS (MKRN2 opposite strand protein) ubiquitin E3-protein ligase taking part in regulating NF-κB-mediated inflammatory responses (201), DESI1 desumolyase (96), DESI2 (PPPDE1) deubiquitinase (202), TMEM222 (transmembrane protein 222, C1orf160) of unknown function localizing to the brain (182), as well as LRAT transferring acyl group to retinol (203), LRAT1 with functions related to cell morphology and motility (204), and PLAT, which releases fatty acids from glycerophospholipids (205) and degrades cellular organelles during lens development (206).

Within cluster III are grouped VASH1 and VASH2 detyrosinases important for angiogenesis, axon formation, and mitotic spindle dynamics (207, 208), menin involved in transcriptional network regulation (45), ARY1 and ARY2 detoxificating acetyltransferases (123), NGLY1 deglycosylating glycoproteins for degradation (209), NTAQ1 deamidase, CEP76 centrosomal protein limiting centriole duplication (210), and DRC7—a component of the nexin–dynein regulatory complex (211). Closely related to CEP76 and DRC7 are two inactive proteins C2D2A and C2D2B, the former being a part of the tectonic-like complex and is associated with Meckel syndrome (184). Cluster III contains also several proteins from the PF01841 (Transglut_core) family: seven TGM acetyltransferases playing roles in cell signaling, cytoskeleton organization, muscle contraction, inflammation (212, 213), factor XIII stabilizing the fibrin clot (214), KY peptidase is important for muscle functioning (215), and catalytically inactive EPB42 related to hereditary spherocytosis, a chronic nonimmune hemolytic anemia (216). FBX21 (FBXO21) is also an inactive transglutaminase-like domain fused to N-terminal F-box-related (PF15966) and C-terminal YccV-like hemimethylated DNA-binding (PF08755) domains and mediates ubiquitination of the EID1 protein (124). Eventually, armadillo repeat–containing protein 3 contains a papain-like domain lacking catalytic residues; it is a homolog of yeast Vac8 (which retains only N-terminal helical region of TPR repeats) and murine armadillo repeat–containing protein 3 taking part in autophagy regulation (217).

Several catalytically inactive proteins, PAN2 and SNUT2 (cluster IV) and XPC (cluster III), contribute to nucleic acid processing. The papain-like domain of PAN2 plays scaffolding roles within the PAN2–PAN3 deadenylation complex (132), and SNUT2 is essential for U4/U6.U5 tri–snRNP spliceosome (131). XPC interacts with DNA and is a part of the XPC–RAD23B–CETN2 complex recognizing DNA lesions during nucleotide excision repair (106, 107).

Ceroid-lipofuscinosis neuronal protein 5 is an S-depalmitoylase linked to neurodegeneration (218) and takes part in endosomal sorting (219).

### Relationship to human diseases

According to UniProt-OMIM crossreferences, of 192 human papain–like proteins (Table S2), 44 are linked to multiple genetic diseases, including neurodegenerative (*e.g.*, UCHL1—Parkinson disease 5 [MIM: 613643], ATX3—spinocerebellar ataxia [MIM: 109150], ALG13—encephalopathy [MIM: 300884], USP9X—X-linked intellectual developmental disorder [MIM: 300919]), hematologic (*e.g.*, EBP42—spherocytosis [MIM: 612690], UBP18—pseudo-TORCH syndrome 2 [MIM: 617397], UBP16—myelomonocytic leukemia (220), F13A—factor XIII deficiency [MIM: 613225]), muscular (*e.g.*, muscular dystrophy [MIM: 618129], KY—myopathy [MIM: 617114]), skin (*e.g.*, TGM1—ichthyosis [MIM: 242300], CYLD—cylindromatosis [MIM: 132700], CATC—Papillon–Lefevre syndrome [MIM: 245000]), inflammatory (*e.g.*, CAN5—vitreoretinopathy [MIM: 193235], OTUL—autoinflammation, panniculitis, and dermatosis syndrome [MIM: 617099], TNAP3—autoinflammatory syndrome [MIM: 616744]), skeletal (*e.g.*, CATK—pycnodysostosis [MIM: 265800], UFSP2—Beukes familial hip dysplasia [MIM: 142669]), digestory (TGM2—major autoantigen in celiac disease (221, 222)), or retina-related (*e.g.*, UBP45—Leber congenital amaurosis 19 [MIM: 618513], LRAT—Leber congenital amaurosis 14 [MIM: 613341]) disorders, and tumors (BAP1—malignant mesothelioma [MIM: 156240], UBP8—pituitary adenoma [MIM: 219090], menin—endocrine neoplasia [MIM: 131100]). Calpain10 is related to diabetes mellitus (MIM: 601283). For a complete list, please refer to Table S2.

## Discussion

Using transitive homology detection searches started with the initial set of known papain-like protein families, we classified 105 Pfam, 89 MEROPS, 25 COG, and 47 KOG protein families as well as 192 human and multiple PDB representatives as related to papain.

**Figure 6:**
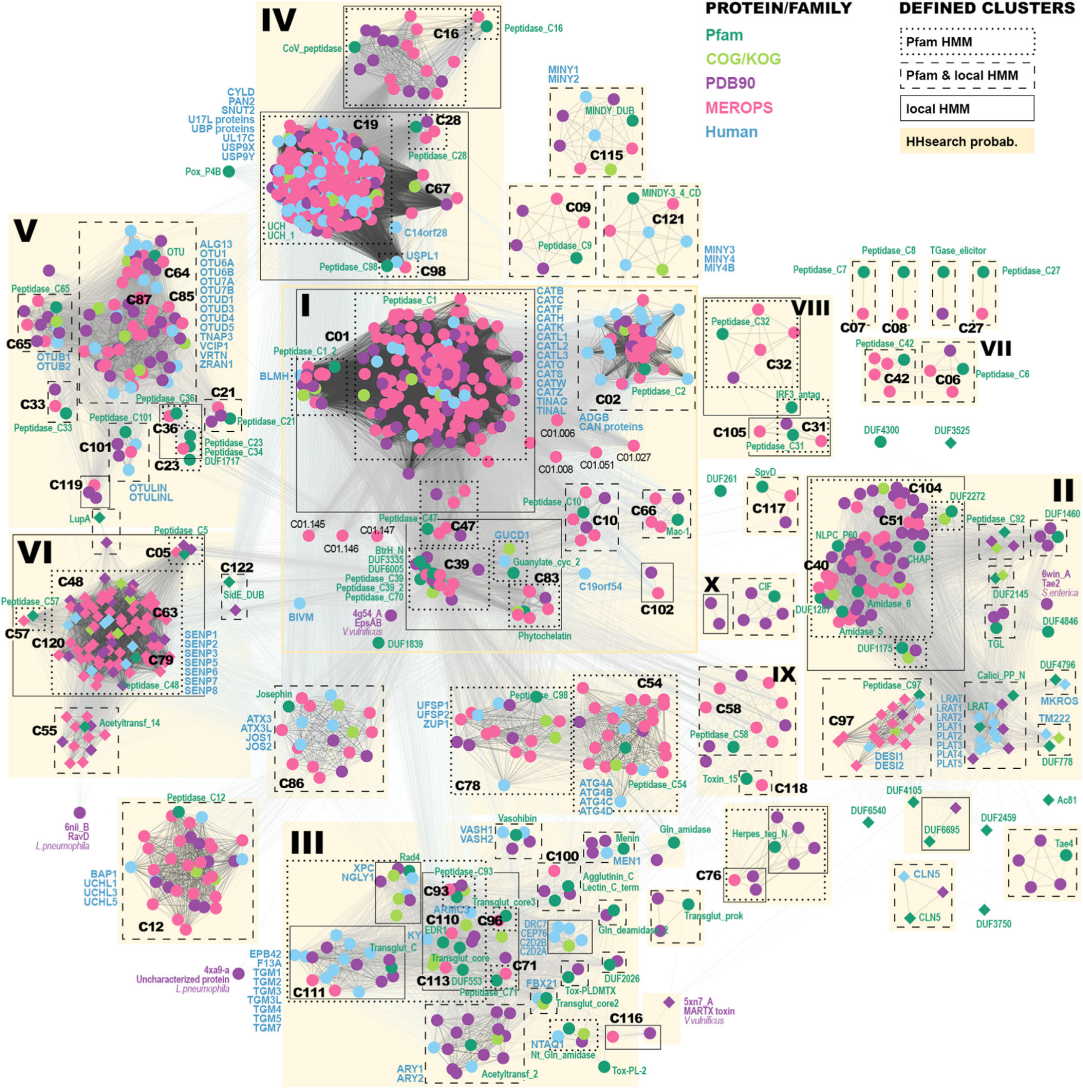
**Schematic view on the sequence space of papain-like cysteine peptidases.** Proteins/families with the core α-helix permuted are marked with *diamonds*. Sequence similarities are represented with *lines* colored according to HHsearch probability score. *Dotted rectangles* mark proteins clustered within given Pfam family based on Pfam-derived HMM profiles; *dashed rectangles* group proteins clustered within family based on both Pfam-derived and locally computed HMM profiles (the latter using E-value threshold of 1E-13); *solid rectangles* mark proteins clustered together only using locally calculated HMM profiles. *Yellowish areas* highlight clusters obtained based on HHsearch mappings; clusters containing more than one Pfam family are indexed with *roman numerals*.

One of the ways to understand similarities between proteins is to group them into families of closely related homologs, and further, into superfamilies of remotely homologous proteins sharing structural features rather than easily detectable sequence similarity. Here, Pfam provides a convenient database of HMM profiles for instant scanning of the sequence of interest against defined families assembled into clans/superfamilies. Pfam profiles encode curated statistical measures for deciding whether the scanned sequence belongs to the family. However, those profiles obviously are computed for Pfam entries only, and if one would like to obtain sequence-to-profile mappings against another database, then HMM profiles need to be computed separately. Hence, we prepared HMM profiles for each Pfam, COG/KOG, PDB90, MEROPS, and human proteins and used them for further hmmscan mappings to generate 89 clusters of closely related protein families. Generally, user-generated HMM profiles are less specific than Pfam derived but still connect functionally related protein families (Fig. 6 and Table S1). Furthermore, based on HHsearch mappings reflecting more remote sequence similarities, we defined 10 major clusters, each containing more than two Pfam families. Those clusters remain functionally and structurally consistent, demonstrating that such similarities remain reflected in protein sequences and are recognized by the fold recognition approach (please note conservation of structural elements within the clusters presented in Fig. 2).

Since some known peptidase families contain proteins performing multiple functions not necessarily connected to peptide bond cleavage, a famous example of which might be the S33 family of serine peptidases, one might object against the application of remote homology detection methods for searching for new families bearing peptidase activity. Known proteins retaining the “cysteine proteinases” fold are classified as peptidases, acetyltransferases, and deamidases, which are overlapping categories of enzymes processing chemically similar bonds, although in different biological contexts. Their families also barely overlap regarding sequence similarity, which allows to expect that performed functions would also remain different. On the other hand, the families of α/β hydrolases, one of which is S33, are much more closely related according to contemporary sequence similarity measures, and in order to provide descriptive rationales for their discrimination, different approaches should be used. Eventually, we believe that the structure and function assignments proposed in this work need experimental confirmation.

Papain-like cysteine peptidases display a remarkable diversity in sequence and structure. Extensive insertions to the structural core, catalytic site migrations, as well as spatial deterioration of its elements hinder systematic analysis of this superfamily with the use of known protein structures. Even more, observed multiple cases of structural permutations might question the definition of the commonly shared structural fold. On the other hand, insisting on the definition of the fold, the assignment of many protein families to the superfamily would be doubtful. However, despite observed variations within the papain-like structural core, all the elements still superimpose well and maintain the general shape of functional peptidase domain, not to mention that permuted β-strands may still carry catalytic residue. Although we cannot judge whether all these proteins share a common ancestor or rather emerged independently converging to the observed forms, we can still opt for demonstrating their clear structural and functional resemblance.

The catalytic dyad–triad, although conserved within the papain-like structures, is often separated in protein sequences by tens to hundreds of residues. The core α-helix harboring catalytic cysteine may be connected to the rest of the peptidase structural core through extensive insertions shaping the enzyme's specificities and biological functions. This, together with permutations and active-site migrations altering the expected order of catalytic residues, disrupts sequence signature characteristics for papain-like enzymes and hampers the identification of new members of this superfamily. Homology detection methods require sequence fragment or amino acid signature, based on which they might expand the alignment and assess the similarity by means of statistical model they implement. For enzymes, the motifs might reflect, that is, active site residues. When such an ephemeral signal dissolves within sequence and structure rearrangements, homology detection becomes a challenging task requiring to consider additional protein features such as predicted secondary structure elements, conservation of hydrophobic residues, as well as to anticipate potential active-site variations. On top of the structural diversity, it might be an additional obstacle for annotating already known proteins as well as for planning or interpreting experimental studies focusing on the molecular biology of processes related to papain-like peptidases.

Especially intriguing and poorly explored is substitution of cysteine residue with serine. Although both might play the same roles in catalysis, their physicochemical differences would require different conditions for the catalysis to proceed (48). With expanding papain-like cysteine proteinases superfamily, even more families occur to have conserved serine instead of cysteine, which might suggest that it is rare but, nevertheless, observed phenomenon.

Observed structural and functional differences within papain-like cysteine proteinases correlate with our clustering based on profile–profile mappings. Not only papain, cathepsins, and multiple virulence factors (cluster I) but also deubiquitinases and viral accessory peptidases, including the one from coronaviruses (cluster IV) retain canonical structures, with well-preserved catalytic triads and oxyanion hole. Deubiquitinases have an additional characteristic “fingers” domain for their substrate binding. Cluster V also groups deubiquitinating enzymes and viral polyprotein peptidases, but they have strand β1' reversed in direction, strand β5 reversed and permuted, and strands β3 and β4 permuted. Moreover, some families retain as little as α-helix and strands β1 and β2 only. NlpC/P60, CHAP, and amidases from cluster II have shorter strand β1' and share characteristic “GD” motif located on strand β1. Acetyltransferases, mostly deamidases and transglutaminases functioning as toxic virulence factors (cluster III), almost always lack structural element harboring oxyanion hole, are devoid of strand β1', and display high variability within strands β4 and β5. A clearly separate group is formed by families with permuted α-helix. These include deubiquitinases and desumolyases from clusters II and VI. Interestingly, cluster II contains both canonical and permuted sequences, which might point at relatively recent divergence.

Although human proteome is well characterized, we were still able to newly identify papain-like domains within. Many papain-like proteins are connected to developing phenotypes in multiple genetic disorders, including neurodegenerative, blood-, skin-, or skeleton-related, as well as tumors. Providing structural bases of the function of proteins involved might facilitate understanding of these, in many cases, severe conditions, and open new tracks of research. Eventually, developing a systematic map of papain-like superfamily should highlight structurally and functionally understudied families and inspire further research aiming at elucidating their specific functions.

## Experimental procedures

Known papain-like cysteine peptidase families and structures were collected from MEROPS, version 12.4 (223), Pfam, version 35 (224), and PDB (downloaded on January 30, 2022) (225) databases and were used as queries for further HHsearch (226) searches (HHsuite, version 3.3.0) against a local database of sequence profiles precomputed for the whole MEROPS, Pfam, PDB90 (PDB SEQRES database clustered to 90% of sequence identity to reduce redundancy) and human proteome (reference proteome derived from the UniProt database). HHsuite contains multiple top-performing profile–profile comparison programs, which efficiently detect similarities between protein sequences with decent sensitivity and selectivity. Collected sequences were mapped to Pfam families using the pfam_scan script (based on HMMER3 (227)) provided with Pfam database. For Pfam families without known structures, AlphaFold2 (228) models were calculated for representative proteins (identifiers of modeled proteins are provided in Fig. 2) using ColabFold (229) or derived from the UniProt database if available. AlphaFold2 is a protein structure prediction method successfully combining information derived from sequence profiles and sophisticated neural network analyses.

To study sequence conservation and taxonomic distribution, homologs belonging to the identified families were collected using National Center for Biotechnology Information (NCBI) PSI-BLAST (230) searches (five iterations, inclusion threshold 1E-5, query coverage 80%) against local nr database (downloaded on July 21, 2022), clustered with CD-HIT (231) and aligned with MAFFT (232). CD-HIT is a simple but reliable and efficient sequence clustering program, and MAFFT is one of the most accurate multiple sequence alignment programs combining multiple measures for maximizing result's consistency. Secondary structures for representative sequences were predicted using PSI-PRED (233). The genomic context for chosen bacterial families was inspected based on gene descriptions from GenBank (234) and using NCBI's E-utilities for deriving neighboring genes. Taxonomic assignments come from the NCBI taxonomy database downloaded together with nr.

All PDB90 structures and 3D models of papain-like proteins were superimposed using SPDBV (235), a convenient 3D viewer offering multiple tools for structure analysis and manipulation. Structure-based multiple sequence alignment between all identified families was manually optimized for a functional fit and corrected for active-site overlap. At this stage, AlphaFold2 models were in addition compared with HHsearch mappings to check prediction consistency (by visually comparing structural alignment between the AlphaFold2 model and the most similar protein of known structure, with the corresponding sequence alignment from HHsearch) and assessed according to conservation of catalytic residues, and core structural elements.

In order to explore evolutionary relationships between identified families, collected Pfam, MEROPS, human, and PDB90 representative sequences were clustered using MCL (236) in Cytoscape's clusterMaker app (237), based on pairwise HMMER3 hmmscan (E-value cutoff 1E-13, chosen arbitrarily from the range where the clustering did not change with the threshold) (227). hmmscan scans a given sequence against precalculated library of HMM profiles (*e.g.*, Pfam database), which boosts sensitivity compared with regular sequence-sequence comparison. MCL is an unsupervised graph clustering algorithm based on flow simulation in networks (236). Furthermore, for more distant evolutionary relationships, the second MCL clustering was performed, this time based on HHsearch mappings (threshold: probability score 80%).

## Data availability

All tables summarizing described results are provided as supporting information to this article.

## Supporting information

This article contains supporting information (5, 7, 8, 9, 10, 11, 12, 13, 14, 23, 24, 27, 29, 35, 36, 37, 38, 40, 41, 42, 43, 45, 50, 51, 52, 53, 54, 55, 58, 59, 64, 69, 71, 76, 95, 96, 97, 98, 99, 101, 105, 109, 111, 112, 120, 121, 122, 123, 125, 126, 136, 137, 138, 140, 141, 142, 143, 144, 146, 147, 148, 150, 151, 152, 153, 154, 159, 160, 161, 164, 166, 167, 168, 169, 172, 174, 175, 176, 177, 178, 179, 180, 181, 182, 183, 184, 193, 197, 198, 201, 210, 211, 218, 238, 239, 240, 241, 242, 243, 244, 245, 246, 247, 248, 249, 250, 251, 252, 253, 254, 255, 256, 257, 258, 259, 260, 261, 262, 263, 264, 265, 266, 267, 268, 269, 270, 271, 272, 273, 274, 275, 276, 277, 278, 279, 280, 281, 282, 283, 284, 285, 286).

## Conflict of interest

The authors declare that they have no conflicts of interest with the contents of this article.
