# Supplementary material for: Identification and classification of papain-like cysteine proteinases
Source: J Biol Chem. 2023 May 8;299(6):104801. doi: 10.1016/j.jbc.2023.104801 (PMC10318531; doi:10.1016/j.jbc.2023.104801)
Supplement: Table S1 [file mmc1.docx]

Table S1 Clusters of papain-like cysteine proteinases defined in this study.

| **Cluster** | **Group** | **MEROPS Clan** | **MEROPS Family** |  |  | **Taxonomy** | | | | |  |
| --- | --- | --- | --- | --- | --- | --- | --- | --- | --- | --- | --- |
|  |  |  |  | **Family / protein name** | **PFAM/CDD/ PDB90/HUMAN** | **Bacteria** | **Archaea** | **Eukaryota** | **Viruses** | **Human** | **Function** |
| I | 1 | CA | C01 C47 | Peptidase C1 Peptidase C1-like PepC Bleomycin hydrolase Cathepsin | PF00112 PF03051 COG3579 COG4870 KOG1542 KOG1543 KOG1544 KOG4128 5jt8_A | 21625 | 1428 | 24473 | 181 | 14 | Protein recycling and homeostasis, cellular turnover; typically lysosomal or secreted [pmid:32228244][pmid:17825974][pmid:15195995] |
| I | 2 |  | **NEW** | **BIVM; Basic immunoglobulin-like variable motif-containing protein** | **Q86UB2** | 6 |  | 174 |  | 1 | Unknown |
| I | 3 |  |  | EpsAB, General Secretion Pathway ProteinType 2 Secretion System protein | 4g54_A | 3103 | 4 | 5 |  |  | Type II secretion system component [pmid:23385451] |
| I | 4 | CA | C02 | Peptidase_C2 Calpain | PF00648 KOG0045 1kfu_L | 1979 | 26 | 9547 | 1 | 15 | Non-degradative, calcium-dependent proteases involved in diverse physiological funcitions, e.g. involved in cytoskeletal remodeling and signal transduction, proteasome-independent degradation [pmid:21531719][pmid:23035980] |
| I | 5 | CA | C54 | Peptidase_C54 Apg4p/Aut2p | PF03416 KOG2674 2d1i_A |  | 1 | 3225 | 1 | 4 | Maturation, de-conjugation from the lipids and recycling of proteins involved in autophagosome formation [pmid:16325851][pmid:16183633] |
| I | 6 | CA | C78 | Peptidase_C78 | PF07910 KOG2433 KOG4696 2z84_A | 3 |  | 2815 |  | 3 | Maturation and deconjugation of ubiquitin-like proteins [pmid:18321862][pmid:29576527][pmid:17182609] |
| I | 7 | CA | C66 | Peptidase_C66 IdeS | PF09028 1y08_A | 854 | 10 | 6 |  |  | Degradation of IgG and blocking opsonophagocytosis [pmid:15574492][pmid:12438365] |
| I | 8 |  | **NEW** | **DUF1839** | **PF08893** | 806 | 1 | 1 |  |  | Fatty acid/phospholipid protein processing (predicted) |
| I | 9 |  | **NEW** | **DUF261** | **PF03196** | 227 | 10 | 6 | 16 |  | Unknown |
| I | 10 | CA | C10 | Peptidase_C10 | PF01640 3bb7_A | 9933 | 42 |  |  |  | Bacterial virulence factors, e.g. Interpain A [pmid:17993455] or SpeB [pmid:32662975] |
| I | 11 | CA | C39 C39_2 C47 C70 C83 **NEW** | Peptidase_C39 Phytochelatin Peptidase_C47 Guanylate_cyc_2 DUF3335 Peptidase_C70 Peptidase_C39_2 BtrH_N **DUF6005** SunT YvpB Staphopain Phytochelatin synthase | PF03412 PF05023 PF05543 PF09778 PF11814 PF12385 PF13529 PF14399 **PF19468** COG2274 COG3271 COG4990 KOG0632 KOG4621 1x9y_D | 69717 | 1415 | 1815 | 219 | 1 | Virulence [pmid:3422637][pmid:23235402][pmid:30928616], defense [pmid:20851126][pmid:11132962], regulation [pmid:24915571], removing peptides from proteins during translocation [pmid:30638446][pmid:28264013], siderophore biosynthesis (predicted), synthesis of phylochelatins [pmid:16339904] |
| I | 12 | CA | C102 | GtgERab GTPase | 5oed_A | 5 |  |  |  |  | Virulence factor [pmid:29298974] |
| I | 13 |  |  | C19orf54 | Q5BKX5 | 34 |  | 630 |  | 1 | Actin maturation [pmid: 36173861] |
|  | 14 |  |  | RavD | 6nii_B | 13 |  |  |  |  | Bacterial effector deubiquitinase [pmid:31110362] |
| III | 15 |  |  | Tox-PL-2 | PF15643 | 469 | 1 |  |  |  | Toxin, transglutaminase [pmid:22731697] |
| III | 16 |  |  | Vasohibin | PF14822 6jzc_A |  | 1 | 335 |  | 2 | Angiogenin regulation [pmid:31908845][pmid:31270470], microtubule detyrosination [pmid:31324789] |
| III | 17 |  |  | Tox-PLDMTX | PF15645 4g29_A | 159 |  | 1 |  |  | Toxin, transglutaminase [pmid:23151626] |
| III | 18 |  |  | DUF2026 | PF09641 2hly_A | 265 |  |  |  |  | Probable virulence factor |
| III | 19 | U | C116 | Dermonecrotic toxin | 2ebf_X | 1 |  |  |  |  | Virulence factor [pmid:17360394] |
| III | 20 |  |  | Menin | PF05053 3u84_A |  |  | 180 |  | 1 | Transcriptional networks regulation [pmid:16415155][pmid:22327296][pmid:28782520][pmid:35534777][pmid:23850066][pmid:22327296] |
| III | 21 |  |  | Acetyltransf_2 NhoA | PF00797 COG2162 4guz_A | 9609 | 79 | 2739 | 14 | 2 | Detoxification, acetyltransferase [pmid:25372695] |
| III | 22 | CA | C71 C93 C96 C110 C111 C113 | Transglut_core DUF553 Peptidase_C93 Peptidase_C71 Transglut_core3 EDR1 YebA CYK3 TGc Coagulation factor XIII | PF01841 PF04473 PF06035 PF12386 PF13471 PF14381 COG1305 COG1800 COG3672 COG5279 KOG4575 4u65_E | 121634 | 4191 | 6999 | 20 | 11 | Post-translational protein modification via transamidase, acetylase, or hydrolase activity [pmid:15288868], tissue transglutaminase [pmid:15069073], virulence factor [pmid:26483615][pmid:31377195][pmid:26085092][pmid:10198038], type II secretion system protein [pmid:22466878], blood clothing [pmid:24115223] |
| III | 23 |  |  | Transglut_core2 SirB1 FBXO21 | PF13369 COG2912 | 8319 | 108 | 495 |  | 1 | Ubiquitylation and proteasomal degradation [pmid:26085330], virulence factor [pmid:26085092] |
| III | 24 |  |  | Rad4 Peptide:N-glycanase Nucleotide excision repair complex XPC-HR23B | PF03835 COG5535 KOG0909 KOG2179 3esw_A | 109 | 105 | 3700 |  | 2 | Nucleotide excision repair, inactive [pmid:34099686][pmid:11487565], misfolded glycoproteins de-glycosylation [pmid:18854368] |
| III | 25 |  |  | Gln_deamidase_2 | PF18626 3a54_A | 741 | 3 | 1 |  |  | Type IVb secretion system effector [pmid:29354599], protein transglutaminase [pmid:21926168] |
| III | 26 |  |  | Nt_Gln_amidase | PF09764 KOG3261 4w79_A | 198 | 1 | 1103 | 1 | 1 | Protein degradation, transglutaminase [pmid:25356641] |
| III | 27 | CA | C100 | Agglutinin C-terminal Ricin-type beta-trefoil lectin C-terminal domain | PF18022 PF18021 5mu9_A | 200 | 98 | 146 | 5 |  | Defense [pmid:28665586], unknown [pmid:19426740] |
| III | 28 |  |  | Gln_amidase | PF15644 | 2022 |  |  | 189 |  | Toxin [pmid:22731697], glutamine deamidase [pmid:22407319] |
| III | 29 |  |  | ORF169b | 3b21_A | 6 |  |  |  |  | Inflammatory response dempening, glutamine deamidase [pmid:22407319][pmid:23542009] |
| III | 30 |  | **NEW** | **C2D2A C2D2B CEP76 DRC7** **C2 Ca2+-binding motif-containing protein** | **Q8IY82 Q8TAP6 Q9P2K1 Q6DHV5 KOG3639** | 204 | 88 | 1073 |  | 4 |  |
| II | 31 |  |  | DUF1460 | PF07313 4q5k_A | 4820 | 5 | 2 | 1 |  | Peptidoglycan degradation, amidase [pmid:25465128] |
| II | 32 | CO CA | C40 C51 C104 | NLPC_P60 CHAP Amidase_5 DUF1175 DUF1287 DUF2272 Amidase_6 | PF00877 PF05257 PF05382 PF06672 PF06940 PF10030 PF12671 COG0791 COG3234 COG3738 COG3942 COG4322 3npf_A | 115951 | 202 | 683 | 1817 |  | Virulence effector [pmid:23730712][pmid:32176738][pmid:23043507], interaction with peptidoglycan [pmid:34115884], peptidoglycan degradation, amidase [pmid:23184858][pmid:27934875], cell wall remodelling [pmid:21864539][pmid:30969170], trypanothione biosynthesis [pmid:18420578], plant cell wall degradation in herbivores' rumen [pmid:23457513], type VI effector [pmid:22750141] |
| II | 33 |  | **NEW** | **DUF4846** | **PF16138** | 2330 | 1 | 1 |  |  | Unknown |
| II | 34 |  |  | TGL | PF20085 4p8i_A | 996 | 5 |  |  |  | Spore cell wall hardening, protein-cross linking [pmid:26322858] |
| II | 35 |  |  | Type 6 secretion amidase effector 2 | 6win_A | 539 | 5 | 131 |  |  | Type 6 secretion amidase effector, cell wall-degradation [pmid:33306955] |
|  | 36 |  | **NEW** | **DUF4300** | **PF14133** | 483 |  |  | 2 |  | Unknown |
|  | 37 | CA | C86 | Josephin Ataxin 3 | PF02099 KOG2934 KOG2935 3o65_G | 2 | 3 | 1864 |  | 4 | Deubiquitination in ubiquitin-proteasome pathway, structure/motility, and signal transduction [pmid:17234717][pmid:16118278][pmid:15767577][pmid:21118805][pmid:23625928] |
| IV | 38 | CA | C19 C28 C67 C98 **NEW** | UCH UCH_1 Ubiquitin-specific protease Peptidase_C28 Peptidase_C98 **C14orf28** | PF00443 PF05408 PF13423 PF15499 COG5077 COG5207 COG5533 COG5560 KOG0944 KOG1275 KOG1863 KOG1864 KOG1865 KOG1866 KOG1867 KOG1868 KOG1870 KOG1871 KOG1872 KOG1873 KOG1887 KOG2026 KOG3556 KOG4598 **Q4W4Y0** 2vhf_A | 323 | 15 | 41903 | 186 | 77 | Deubiquitination [pmid:19626045][pmid:16325574][pmid:33795880][pmid:17035239][pmid:28945249][pmid:20395473], structural scaffolding in tri-snRNP complex [pmid:26912367], viral virulence [pmid:9857201] |
| IV | 39 | CA | C16 | Peptidase_C16 CoV_peptidase | PF01831 PF08715 3mp2_A |  |  |  | 2078 |  | Viral accessory peptidase, deubiquitinating activity [pmid:16581910][pmid:16306590] |
|  | 40 |  | **NEW** | **Pox_P4B** | **PF03292** | 4 |  |  | 67 |  | Viral core protein [pmid:8291244] |
|  | 41 | CA | C12 | Peptidase_C12 UCHL1 | PF01088 KOG1415 KOG2778 1xd3_A | 2 | 2 | 6144 |  | 4 | Deubiquitinase [pmid:12485996][pmid:19188440][pmid:19117993] |
|  | 42 | CA | C76 | Herpes_teg_N | PF04843 2jyq_A | 15 |  | 1120 | 92 |  | Viral cycle, deubiquitinase [pmid:17349955][pmid:16306630][pmid:18216103][pmid:20190741][pmid:19923173] |
| X | 43 |  |  | CIF | PF16374 3eir_A | 53 |  |  |  |  | Virulence, ubiquitin deamidase, type III secreted effector [pmid:22069713][pmid:18705694] |
| X | 44 |  |  | MavC | 7bxf_A | 18 |  |  |  |  | Virulence, deubiquitinase, deamidase [pmid:32488130] |
|  | 45 | CA | C117 | SpvD | PF05563 5lq6_A | 18 |  |  |  |  | Virulence, deubiquitinase [pmid:27789710][pmid:27232334] |
|  | 46 |  |  | Transglut_prok | PF09017 3iu0_A | 121 |  |  |  |  | Defence, transglutaminase [pmid:28751378][pmid:30318745] |
|  | 47 |  |  | Tae4 | PF14113 4jur_A | 1842 |  | 72 |  |  | Type VI secretion system [pmid:23288853], peptidoglycan hydrolysis [pmid:30511676] |
|  | 48 |  |  | TGase_elicitor | PF16683 3tw5_A | 1017 | 5 | 226 |  |  | Transglutaminase [pmid:21994936] |
| VIII | 49 | CA | C31 C105 | Peptidase_C31 | PF05410 3ifu_A |  |  |  | 159 |  | Virulence [pmid:23287061][pmid:27881655][pmid:18198380][pmid:19706710] |
| VIII | 50 | CA | C32 | Peptidase_C32 IFR3_antag | PF05411 PF14754 3mtv_A |  |  |  | 540 |  | Virulence, deubiquitinase [pmid:18198380][pmid:28235682][pmid:18078692] |
| IX | 51 | U | C118 | Toxin_15 | PF07906 | 248 |  |  |  |  | Type 3 secretion system effector [pmid:32657447] |
| IX | 52 |  |  | PatoxP | 6hv6_A | 800 |  | 23 |  |  | Virulence, deamidase [pmid:30478175] |
| IX | 53 | CA | C58 | Peptidase_C58 | PF03543 1ukf_A | 197 |  |  |  |  | Virulence [pmid:11952132] [pmid:17277084] |
|  | 54 | CA | C121 | MINDY-3_4_CD | PF13898 KOG2871 |  | 1 | 1789 |  | 3 | Deubiquitinase [pmid:27292798] |
|  | 55 | CA | C115 | MINDY_DUB | PF04424 KOG2427 5jkn_A | 1 |  | 1785 |  | 2 | Deubiquitinase [pmid:27292798][pmid:28082312] |
|  | 56 | U | C07 | Peptidase_C7 | PF01830 |  |  | 1 | 14 |  | Viral acessory peptidase [pmid:1853573] |
| VII | 57 | U | C42 | Peptidase_C42 | PF05533 |  |  |  | 55 |  | Viral acessory peptidase [pmid:11711606][pmid:30650571], transport [pmid:12584307] |
| VII | 58 | CA | C06 | Peptidase_C6 | PF00851 3rnv_A |  |  |  | 348 |  | Viral acessory peptidase, transport [pmid:11414807] |
|  | 59 | CN | C09 | Peptidase_C9 | PF01707 4gua_A | 1 |  |  | 52 |  | Virulence [pmid:23010928] |
| V | 60 | U | C23 C36 | Peptidase_C23 Peptidase_C34 Peptidase_C36 | PF05379 PF05413 PF05415 | 5 |  |  | 388 |  | Virulence [pmid:7871721][pmid:11711606][pmid:9191870] |
| V | 61 | U | **NEW** | **DUF1717** | **PF05414** |  |  |  | 13 |  | Virulence |
| V | 62 | CA | C21 | Peptidase_C21 | PF05381 5lwa_B |  |  | 1 | 130 |  | Virulence, deubiquitinase [pmid:29117247] |
| V | 63 | CA | C64 C85 C87 **NEW** | OTU NF-kappa B regulator AP20/Cezanne **Vertnin** | PF02338 COG5539 KOG2605 KOG2606 KOG3288 KOG4345 5lrx_A **Q9H8Y1** | 282 | 7 | 12013 | 144 | 13 | Signalling, deubiquitinase [pmid:10664582][pmid:18164316][pmid:27732584] |
| V | 64 | CA | C65 | Peptidase_C65 | PF10275 KOG3991 4dhz_A | 9 | 1 | 2265 |  | 3 | Deubiquitinase [pmid:22367539][pmid:25590432] |
| V | 65 | CA | C101 | Peptidase_C101 | PF16218 3znv_A |  |  | 572 |  | 2 | Deubiquitinase [pmid:23708998][pmid:23746843] |
| V | 66 | CA | C33 | Peptidase_C33 | PF05412 4ium_A |  |  |  | 496 |  | Viral acessory peptidase, deubiquitinase [pmid:8617757][pmid:23401522] |
| V | 67 |  |  | Ceg23 | 6ks5_A | 37 |  |  |  |  | Virulence, deubiquitinase [pmid:31907282] |
| V | 68 | CA | C119 | LotA | 7bu0_A | 109 |  |  |  |  | Virulence, deubiquitinase [pmid:33136002][pmid:34633867] |
|  | 69 | U | C27 | Peptidase_C27 | PF05407 |  |  |  | 7 |  | Viral acessory protein [pmid:10823845][pmid:17475644] |
|  | 70 |  |  | Uncharacterized protein | 4xa9-a | 6 |  |  |  |  |  |
|  | 71 |  |  | CLN5 | PF15014 6r99_A |  |  | 292 | 1 | 1 | Homeostatis, S-depalmitoylase [pmid:35427157] |
|  | 72 |  | **NEW** | **DUF2459** | **PF09601** | 4045 |  | 2 | 1 |  | Urease biosynthesis (predicted) |
| II | 73 |  | **NEW** | **DUF4796** | **PF16044** |  |  | 731 |  | 1 | E3 ubiquitin-protein ligase [pmid:28378844] |
| II | 74 |  | **NEW** | **DUF6540** | **PF20174** |  |  | 2845 |  |  | Unknown |
| II | 75 | CP | C97 | Peptidase_C97 | PF05903 KOG0324 3ebq_A | 6 | 3 | 4788 | 3 | 2 | deSUMOylation [pmid:22370726][pmid:22498933], export [pmid:29666234] |
| II | 76 |  | **NEW** | **DUF778** | **PF05608 KOG3150** |  | 1 | 1087 |  | 1 | Unknown function related to the functioning of membrane receptors [pmid:33824500][pmid:18643990] |
| II | 77 |  |  | LRAT Calici_PP_N | PF04970 PF08405 4dpz_X | 3430 |  | 5339 | 112 | 8 | Acetyltransferase [pmid:22605381][pmid:20628054], phospholipase [pmid:22290676][pmid:25383759] |
| II | 78 |  | **NEW** | **DUF2145** | **PF09916 COG4727** | 1641 |  |  |  |  | Unknown |
| II | 79 |  |  | Peptidase_C92 YycO | PF05708 COG3863 2if6_A | 11059 | 163 | 206 | 90 |  | Pathogen-host interaction, amidase cleaving a bond between lipid and peptide [pmid:21799766] |
| II | 80 |  | **NEW** | **DUF4105** | **PF13387** | 8059 | 7 | 3 |  |  | Related to membrane protein/lipids processing (predicted) |
| II | 81 |  | **NEW** | **DUF6695** TseH | **PF20405** 6v98_A | 803 |  |  | 1 |  | Virulence [pmid:32094588] |
|  | 82 |  | **NEW** | **DUF3525** | **PF12039** |  |  |  | 35 |  | Polyprotein peptidase (predicted) |
| VI | 83 | CE | C05 C48 C57 C63 C79 C120 | Peptidase_C5 Peptidase_C48 Peptidase_C57 Ulp1 | PF00770 PF02902 PF03290 COG5160 KOG0778 KOG0779 KOG3246 4wx4_A | 212 | 14 | 7784 | 248 | 7 | Viral peptidase [pmid:25571794][pmid:22791715][pmid:32075933], deconjugation of ubiquitin-like modifications [pmid:18799455][pmid:15567417][pmid:27425412] |
| VI | 84 | CE | C55 | Acetyltransf_14 | PF03421 6be0_A | 325 |  |  |  |  | Bacterial effector, acetyltransferase [pmid:30025209][pmid:17116858] |
| VI | 85 | CE | C122 | SidE_DUB | PF19049 5cra_A | 47 |  |  |  |  | Bacterial effector, deubiquitinase [pmid:26598703] |
| V/VI | 86 |  |  | LupA | PF18242 5dgg_A | 97 |  | 21 |  |  | Bacterial effector, deubiquitinase [pmid:27986836] |
|  | 87 |  | **NEW** | **Ac81** | **PF05820** |  |  |  | 88 |  | Nucleocapsid assembly [pmid:27212683] |
|  | 88 |  |  | MARTX | 5xn7_A | 66 |  |  |  |  | Toxin [pmid:28060924][pmid:17464284] |
|  | 89 |  | **NEW** | **DUF3750** | **PF12570** | 2444 | 4 |  |  |  | Unknown |
