# Supplementary material for: Identification and classification of papain-like cysteine proteinases
Source: J Biol Chem. 2023 May 8;299(6):104801. doi: 10.1016/j.jbc.2023.104801 (PMC10318531; doi:10.1016/j.jbc.2023.104801)
Supplement: Table S2 [file mmc2.docx]

Table S2 A summary of human papain-like proteinases identified in this study.

| **Cluster** | **Group** | **Uniprot ID** | **Active?** | **Length** | **Pfam** | **Protein name** | **Gene names** | **Related disease** |
| --- | --- | --- | --- | --- | --- | --- | --- | --- |
| I | 1 | Q13867 | 1 | 455 | PF03051 | Bleomycin hydrolase | BLMH |  |
| I | 1 | P07858 | 1 | 339 | PF00112 | Cathepsin B | CTSB CPSB | Keratolytic winter erythema (KWE) (MIM:148370) |
| I | 1 | P53634 | 1 | 463 | PF00112 | Dipeptidyl peptidase 1 | CTSC CPPI | Papillon-Lefevre syndrome (PLS) (MIM:245000) keratosis and severe periodontitis; Haim-Munk syndrome (HMS) (MIM:245010) keratosis and periodontitis; Periodontititis, aggressive, 1 (AP1) (MIM:170650) gingival infections |
| I | 1 | Q9UBX1 | 1 | 484 | PF00112 | Cathepsin F | CTSF | Ceroid lipofuscinosis, neuronal, 13 (Kufs type) (CLN13) (MIM:615362) cognitive decline and motor dysfunction |
| I | 1 | P09668 | 1 | 335 | PF00112 | Pro-cathepsin H | CTSH CPSB |  |
| I | 1 | P43235 | 1 | 329 | PF00112 | Cathepsin K | CTSK CTSO CTSO2 | Pycnodysostosis (PKND) (MIM:265800) deformity of the skull, maxilla and phalanges, osteosclerosis, and fragility of bone |
| I | 1 | P07711 | 1 | 333 | PF00112 | Procathepsin L | CTSL CTSL1 |  |
| I | 1 | O60911 | 1 | 334 | PF00112 | Cathepsin L2 | CTSV CATL2 CTSL2 CTSU UNQ268/PRO305 |  |
| I | 1 | Q5NE16 | 0 | 218 |  | Putative inactive cathepsin L-like protein CTSL3P | CTSL3P CTSL3 |  |
| I | 1 | P43234 | 1 | 321 | PF00112 | Cathepsin O | CTSO CTSO1 |  |
| I | 1 | P25774 | 1 | 331 | PF00112 | Cathepsin S | CTSS |  |
| I | 1 | P56202 | 1 | 376 | PF00112 | Cathepsin W | CTSW |  |
| I | 1 | Q9UBR2 | 1 | 303 | PF00112 | Cathepsin Z | CTSZ |  |
| I | 1 | Q9UJW2 | 0 | 476 | PF00112 | Tubulointerstitial nephritis antigen | TINAG |  |
| I | 1 | Q9GZM7 | 0 | 467 | PF00112 | Tubulointerstitial nephritis antigen-like | TINAGL1 GIS5 LCN7 OLRG2 TINAGL PP6614 PSEC0088 UNQ204/PRO230 |  |
| I | 2 | Q86UB2 | 1 | 503 |  | Basic immunoglobulin-like variable motif-containing protein | BIVM |  |
| I | 4 | Q8N7X0 | 0 | 1667 | PF00648 | Androglobin | ADGB C6orf103 CAPN7L |  |
| I | 4 | P07384 | 1 | 714 | PF00648 | Calpain-1 catalytic subunit | CAPN1 CANPL1 PIG30 | Spastic paraplegia 76, autosomal recessive (SPG76) (MIM:616907) neurodegenerative disorder weakness and spasticity of the lower limbs |
| I | 4 | Q9HC96 | 1 | 672 | PF00648 | Calpain-10 | CAPN10 KIAA1845 | Diabetes mellitus, non-insulin-dependent, 1 (NIDDM1) (MIM:601283) disorder of glucose homeostasis caused by a lack of sensitivity to the body's own insulin |
| I | 4 | Q9UMQ6 | 1 | 739 | PF00648 | Calpain-11 | CAPN11 |  |
| I | 4 | Q6ZSI9 | 1 | 719 | PF00648 | Calpain-12 | CAPN12 |  |
| I | 4 | Q6MZZ7 | 1 | 669 | PF00648 | Calpain-13 | CAPN13 |  |
| I | 4 | A8MX76 | 1 | 684 | PF00648 | Calpain-14 | CAPN14 |  |
| I | 4 | O75808 | 1 | 1086 | PF00648 | Calpain-15 | CAPN15 SOLH | Oculogastrointestinal neurodevelopmental syndrome (OGIN) (MIM:619318) neurodevelopmental disorder characterized by multiple growth deficits |
| I | 4 | P17655 | 1 | 700 | PF00648 | Calpain-2 catalytic subunit | CAPN2 CANPL2 |  |
| I | 4 | P20807 | 1 | 821 | PF00648 | Calpain-3 | CAPN3 CANP3 CANPL3 NCL1 | Muscular dystrophy, limb-girdle, autosomal recessive 1 (LGMDR1) (MIM:253600); Muscular dystrophy, limb-girdle, autosomal dominant 4 (LGMDD4) (MIM:618129) |
| I | 4 | O15484 | 1 | 640 | PF00648 | Calpain-5 | CAPN5 NCL3 | Vitreoretinopathy, neovascular inflammatory (VRNI) (MIM:193235) autoimmune condition of the eye - leading to blindness |
| I | 4 | Q9Y6Q1 | 0 | 641 | PF00648 | Calpain-6 | CAPN6 CALPM CANPX |  |
| I | 4 | Q9Y6W3 | 1 | 813 | PF00648 | Calpain-7 | CAPN7 PALBH |  |
| I | 4 | A6NHC0 | 1 | 703 | PF00648 | Calpain-8 | CAPN8 NCL2 |  |
| I | 4 | O14815 | 1 | 690 | PF00648 | Calpain-9 | CAPN9 NCL4 |  |
| I | 5 | Q8WYN0 | 1 | 398 | PF03416 | Cysteine protease ATG4A | ATG4A APG4A AUTL2 |  |
| I | 5 | Q9Y4P1 | 1 | 393 | PF03416 | Cysteine protease ATG4B | ATG4B APG4B AUTL1 KIAA0943 |  |
| I | 5 | Q96DT6 | 1 | 458 | PF03416 | Cysteine protease ATG4C | ATG4C APG4C AUTL1 AUTL3 |  |
| I | 5 | Q86TL0 | 1 | 474 | PF03416 | Cysteine protease ATG4D | ATG4D APG4D AUTL4 |  |
| I | 6 | Q6NVU6 | 0 | 142 | PF07910 | Inactive Ufm1-specific protease 1 | UFSP1 |  |
| I | 6 | Q9NUQ7 | 1 | 469 | PF07910 | Ufm1-specific protease 2 | UFSP2 C4orf20 | Beukes familial hip dysplasia (BFHD) (MIM:142669) degenerative osteoarthritis of the hip joint; Spondyloepimetaphyseal dysplasia, Di Rocco type (SEMDDR) (MIM:617974) |
| I | 6 | Q96AP4 | 1 | 578 | PF07910 | Zinc finger-containing ubiquitin peptidase 1 | ZUP1 C6orf113 ZUFSP |  |
| I | 11 | Q96NT3 | 1 | 240 | PF09778 | Protein GUCD1 | GUCD1 C22orf13 LLN4 |  |
| I | 13 | Q5BKX5 | 1 | 351 |  | UPF0692 protein C19orf54 | C19orf54 |  |
| III | 16 | Q7L8A9 | 1 | 365 | PF14822 | Tubulinyl-Tyr carboxypeptidase 1 | VASH1 KIAA1036 VASH |  |
| III | 16 | Q86V25 | 1 | 355 | PF14822 | Tubulinyl-Tyr carboxypeptidase 2 | VASH2 VASHL |  |
| III | 20 | O00255 | 0 | 615 | PF05053 | Menin | MEN1 SCG2 | Familial multiple endocrine neoplasia type I (MEN1) (MIM:131100) |
| III | 21 | P18440 | 1 | 290 | PF00797 | Arylamine N-acetyltransferase 1 | NAT1 AAC1 |  |
| III | 21 | P11245 | 1 | 290 | PF00797 | Arylamine N-acetyltransferase 2 | NAT2 AAC2 |  |
| III | 22 | Q5W041 | 0 | 872 | PF14381 | Armadillo repeat-containing protein 3 | ARMC3 |  |
| III | 22 | P16452 | 0 | 691 | PF01841 | Protein 4.2 | EPB42 E42P | Spherocytosis 5 (SPH5) (MIM:612690) hematologic disorder leading to chronic hemolytic anemia |
| III | 22 | P00488 | 1 | 732 | PF01841 | Coagulation factor XIII A chain | F13A1 F13A | Factor XIII subunit A deficiency (FA13AD) (MIM:613225) hematologic disorder characterized by a life-long bleeding tendency |
| III | 22 | Q8NBH2 | 1 | 661 | PF01841 | Kyphoscoliosis peptidase | KY | Myopathy, myofibrillar, 7 (MFM7) (MIM:617114) |
| III | 22 | P22735 | 1 | 817 | PF01841 | Protein-glutamine gamma-glutamyltransferase K | TGM1 KTG | Ichthyosis, congenital, autosomal recessive 1 (ARCI1) (MIM:242300) |
| III | 22 | P21980 | 1 | 687 | PF01841 | Protein-glutamine gamma-glutamyltransferase 2 | TGM2 | major autoantigen in celiac disease (pmid\|9212111) deamidates gliadin which creates an epitope that binds efficiently to HLA-DQ2 and is recognized by gut-derived T-cells |
| III | 22 | Q08188 | 1 | 693 | PF01841 | Protein-glutamine gamma-glutamyltransferase E | TGM3 | Uncombable hair syndrome 2 (UHS2) (MIM:617251) |
| III | 22 | O95932 | 1 | 706 | PF01841 | Protein-glutamine gamma-glutamyltransferase 6 | TGM6 TGM3L | Spinocerebellar ataxia 35 (SCA35) (MIM:613908) |
| III | 22 | P49221 | 1 | 684 | PF01841 | Protein-glutamine gamma-glutamyltransferase 4 | TGM4 |  |
| III | 22 | O43548 | 1 | 720 | PF01841 | Protein-glutamine gamma-glutamyltransferase 5 | TGM5 TGMX | Peeling skin syndrome 2 (PSS2) (MIM:609796) |
| III | 22 | Q96PF1 | 1 | 710 | PF01841 | Protein-glutamine gamma-glutamyltransferase Z | TGM7 |  |
| III | 23 | O94952 | 0 | 628 | PF13369 | F-box only protein 21 | FBXO21 FBX21 KIAA0875 |  |
| III | 24 | Q96IV0 | 1 | 654 | PF03835 | Peptide-N(4)-(N-acetyl-beta-glucosaminyl)asparagine amidase | NGLY1 PNG1 | Congenital disorder of deglycosylation 1 (CDDG1) (MIM:615273) |
| III | 24 | Q01831 | 0 | 940 | PF03835 | DNA repair protein complementing XP-C cells | XPC XPCC | Xeroderma pigmentosum complementation group C (XP-C) (MIM:278720) |
| III | 26 | Q96HA8 | 1 | 205 | PF09764 | Protein N-terminal glutamine amidohydrolase | NTAQ1 C8orf32 WDYHV1 |  |
| III | 30 | Q9P2K1 | 0 | 1620 |  | Coiled-coil and C2 domain-containing protein 2A | CC2D2A KIAA1345 | Meckel syndrome 6 (MKS6) (MIM:612284); Joubert syndrome 9 (JBTS9) (MIM:612285); COACH syndrome 2 (COACH2) (MIM:619111) |
| III | 30 | Q6DHV5 | 0 | 1437 |  | Protein CC2D2B | CC2D2B C10orf130 C10orf131 |  |
| III | 30 | Q8TAP6 | 1 | 659 |  | Centrosomal protein of 76 kDa | CEP76 C18orf9 |  |
| III | 30 | Q8IY82 | 1 | 874 |  | Dynein regulatory complex subunit 7 | DRC7 C16orf50 CCDC135 |  |
|  | 37 | P54252 | 1 | 361 | PF02099 | Ataxin-3 | ATXN3 ATX3 MJD MJD1 SCA3 | Spinocerebellar ataxia 3 (SCA3) (MIM:109150) |
|  | 37 | Q9H3M9 | 1 | 355 | PF02099 | Ataxin-3-like protein | ATXN3L ATX3L MJDL |  |
|  | 37 | Q15040 | 1 | 202 | PF02099 | Josephin-1 | JOSD1 JSPH1 KIAA0063 |  |
|  | 37 | Q8TAC2 | 1 | 188 | PF02099 | Josephin-2 | JOSD2 SBBI54 |  |
| IV | 38 | Q4W4Y0 | 1 | 310 |  | Uncharacterized protein C14orf28 | C14orf28 DRIP1 |  |
| IV | 38 | Q9NQC7 | 1 | 956 | PF00443 | Ubiquitin carboxyl-terminal hydrolase CYLD | CYLD CYLD1 KIAA0849 HSPC057 | Cylindromatosis, familial (FCYL) (MIM:132700); Multiple familial trichoepithelioma 1 (MFT1) (MIM:601606); Brooke-Spiegler syndrome (BRSS) (MIM:605041); Frontotemporal dementia and/or amyotrophic lateral sclerosis 8 (FTDALS8) (MIM:619132) |
| IV | 38 | Q504Q3 | 0 | 1202 | PF13423 | PAN2-PAN3 deadenylation complex catalytic subunit PAN2 | PAN2 KIAA0710 USP52 |  |
| IV | 38 | Q53GS9 | 0 | 565 | PF00443 | U4/U6.U5 tri-snRNP-associated protein 2 | USP39 CGI-21 HSPC332 PRO2855 |  |
| IV | 38 | Q7RTZ2 | 1 | 530 | PF00443 | Ubiquitin carboxyl-terminal hydrolase 17-like protein 1 | USP17L1 USP17L USP17L1P |  |
| IV | 38 | Q6R6M4 | 1 | 530 | PF00443 | Ubiquitin carboxyl-terminal hydrolase 17 | USP17L2 DUB3 USP17 USP17H USP17I USP17J USP17K USP17L USP17M |  |
| IV | 38 | A6NCW0 | 1 | 530 | PF00443 | Ubiquitin carboxyl-terminal hydrolase 17-like protein 3 | USP17L3 USP17B USP17F |  |
| IV | 38 | A6NCW7 | 0 | 530 | PF00443 | Inactive ubiquitin carboxyl-terminal hydrolase 17-like protein 4 | USP17L4 |  |
| IV | 38 | A8MUK1 | 1 | 530 | PF00443 | Ubiquitin carboxyl-terminal hydrolase 17-like protein 5 | USP17L5 |  |
| IV | 38 | Q6QN14 | 1 | 398 | PF00443 | Ubiquitin carboxyl-terminal hydrolase 17-like protein 6 | USP17L6P USP17C USP17D USP17N |  |
| IV | 38 | P0C7H9 | 0 | 530 | PF00443 | Inactive ubiquitin carboxyl-terminal hydrolase 17-like protein 7 | USP17L7 |  |
| IV | 38 | P0C7I0 | 0 | 530 | PF00443 | Inactive ubiquitin carboxyl-terminal hydrolase 17-like protein 8 | USP17L8 |  |
| IV | 38 | C9JJH3 | 1 | 530 | PF00443 | Ubiquitin carboxyl-terminal hydrolase 17-like protein 10 | USP17L10 |  |
| IV | 38 | C9JVI0 | 1 | 530 | PF00443 | Ubiquitin carboxyl-terminal hydrolase 17-like protein 11 | USP17L11 |  |
| IV | 38 | C9JLJ4 | 1 | 530 | PF00443 | Ubiquitin carboxyl-terminal hydrolase 17-like protein 13 | USP17L13 |  |
| IV | 38 | C9J2P7 | 1 | 553 | PF00443 | Ubiquitin carboxyl-terminal hydrolase 17-like protein 15 | USP17L15 |  |
| IV | 38 | D6RBQ6 | 1 | 530 | PF00443 | Ubiquitin carboxyl-terminal hydrolase 17-like protein 17 | USP17L17 |  |
| IV | 38 | D6R9N7 | 1 | 530 | PF00443 | Ubiquitin carboxyl-terminal hydrolase 17-like protein 18 | USP17L18 |  |
| IV | 38 | D6RCP7 | 1 | 530 | PF00443 | Ubiquitin carboxyl-terminal hydrolase 17-like protein 19 | USP17L19 |  |
| IV | 38 | D6RJB6 | 1 | 530 | PF00443 | Ubiquitin carboxyl-terminal hydrolase 17-like protein 20 | USP17L20 |  |
| IV | 38 | D6R901 | 1 | 530 | PF00443 | Ubiquitin carboxyl-terminal hydrolase 17-like protein 21 | USP17L21 |  |
| IV | 38 | D6RA61 | 1 | 530 | PF00443 | Ubiquitin carboxyl-terminal hydrolase 17-like protein 22 | USP17L22 |  |
| IV | 38 | D6RBM5 | 1 | 183 | PF00443 | Putative ubiquitin carboxyl-terminal hydrolase 17-like protein 23 | USP17L23 |  |
| IV | 38 | Q0WX57 | 1 | 530 | PF00443 | Ubiquitin carboxyl-terminal hydrolase 17-like protein 24 | USP17L24 USP17 USP17H USP17I USP17J USP17K USP17L USP17M; USP17L25; USP17L26; USP17L27; USP17L28; USP17L29; USP17L30 |  |
| IV | 38 | O94782 | 1 | 785 | PF00443 | Ubiquitin carboxyl-terminal hydrolase 1 | USP1 |  |
| IV | 38 | Q14694 | 1 | 798 | PF00443 | Ubiquitin carboxyl-terminal hydrolase 10 | USP10 KIAA0190 |  |
| IV | 38 | P51784 | 1 | 963 | PF00443 | Ubiquitin carboxyl-terminal hydrolase 11 | USP11 UHX1 |  |
| IV | 38 | O75317 | 1 | 370 | PF00443 | Ubiquitin carboxyl-terminal hydrolase 12 | USP12 UBH1 USP12L1 |  |
| IV | 38 | Q92995 | 1 | 863 | PF00443 | Ubiquitin carboxyl-terminal hydrolase 13 | USP13 ISOT3 |  |
| IV | 38 | P54578 | 1 | 494 | PF00443 | Ubiquitin carboxyl-terminal hydrolase 14 | USP14 TGT |  |
| IV | 38 | Q9Y4E8 | 1 | 981 | PF00443 | Ubiquitin carboxyl-terminal hydrolase 15 | USP15 KIAA0529 |  |
| IV | 38 | Q9Y5T5 | 1 | 823 | PF00443 | Ubiquitin carboxyl-terminal hydrolase 16 | USP16 MSTP039 | Chronic myelomonocytic leukemia (pmid\|18925961) |
| IV | 38 | Q9UMW8 | 1 | 372 | PF00443 | Ubl carboxyl-terminal hydrolase 18 | USP18 ISG43 | Pseudo-TORCH syndrome 2 (PTORCH2) (MIM:617397) |
| IV | 38 | O94966 | 1 | 1318 | PF00443 | Ubiquitin carboxyl-terminal hydrolase 19 | USP19 KIAA0891 ZMYND9 |  |
| IV | 38 | O75604 | 1 | 605 | PF00443 | Ubiquitin carboxyl-terminal hydrolase 2 | USP2 UBP41 |  |
| IV | 38 | Q9Y2K6 | 1 | 914 | PF00443 | Ubiquitin carboxyl-terminal hydrolase 20 | USP20 KIAA1003 LSFR3A VDU2 |  |
| IV | 38 | Q9UK80 | 1 | 565 | PF00443 | Ubiquitin carboxyl-terminal hydrolase 21 | USP21 USP23 PP1490 |  |
| IV | 38 | Q9UPT9 | 1 | 525 | PF00443 | Ubiquitin carboxyl-terminal hydrolase 22 | USP22 KIAA1063 USP3L |  |
| IV | 38 | Q9UPU5 | 1 | 2620 | PF00443 | Ubiquitin carboxyl-terminal hydrolase 24 | USP24 KIAA1057 |  |
| IV | 38 | Q9UHP3 | 1 | 1055 | PF00443 | Ubiquitin carboxyl-terminal hydrolase 25 | USP25 USP21 |  |
| IV | 38 | Q9BXU7 | 1 | 913 | PF00443 | Ubiquitin carboxyl-terminal hydrolase 26 | USP26 |  |
| IV | 38 | A6NNY8 | 1 | 438 | PF00443 | Ubiquitin carboxyl-terminal hydrolase 27 | USP27X USP22L USP27 | Intellectual developmental disorder, X-linked 105 (XLID105) (MIM:300984) |
| IV | 38 | Q96RU2 | 1 | 1077 | PF00443 | Ubiquitin carboxyl-terminal hydrolase 28 | USP28 KIAA1515 |  |
| IV | 38 | Q9HBJ7 | 1 | 922 | PF00443 | Ubiquitin carboxyl-terminal hydrolase 29 | USP29 |  |
| IV | 38 | Q9Y6I4 | 1 | 520 | PF00443 | Ubiquitin carboxyl-terminal hydrolase 3 | USP3 |  |
| IV | 38 | Q70CQ3 | 1 | 517 | PF00443 | Ubiquitin carboxyl-terminal hydrolase 30 | USP30 |  |
| IV | 38 | Q70CQ4 | 1 | 1352 | PF00443 | Ubiquitin carboxyl-terminal hydrolase 31 | USP31 KIAA1203 |  |
| IV | 38 | Q8NFA0 | 1 | 1604 | PF00443 | Ubiquitin carboxyl-terminal hydrolase 32 | USP32 USP10 |  |
| IV | 38 | Q8TEY7 | 1 | 942 | PF00443 | Ubiquitin carboxyl-terminal hydrolase 33 | USP33 KIAA1097 VDU1 |  |
| IV | 38 | Q70CQ2 | 1 | 3546 | PF00443 | Ubiquitin carboxyl-terminal hydrolase 34 | USP34 KIAA0570 KIAA0729 |  |
| IV | 38 | Q9P2H5 | 1 | 1018 | PF00443 | Ubiquitin carboxyl-terminal hydrolase 35 | USP35 KIAA1372 USP34 |  |
| IV | 38 | Q9P275 | 1 | 1123 | PF00443 | Ubiquitin carboxyl-terminal hydrolase 36 | USP36 KIAA1453 |  |
| IV | 38 | Q86T82 | 1 | 979 | PF00443 | Ubiquitin carboxyl-terminal hydrolase 37 | USP37 KIAA1594 |  |
| IV | 38 | Q8NB14 | 1 | 1042 | PF00443 | Ubiquitin carboxyl-terminal hydrolase 38 | USP38 KIAA1891 |  |
| IV | 38 | Q13107 | 1 | 963 | PF00443 | Ubiquitin carboxyl-terminal hydrolase 4 | USP4 UNP UNPH |  |
| IV | 38 | Q9NVE5 | 1 | 1235 | PF00443 | Ubiquitin carboxyl-terminal hydrolase 40 | USP40 |  |
| IV | 38 | Q3LFD5 | 1 | 358 | PF00443 | Putative ubiquitin carboxyl-terminal hydrolase 41 | USP41 |  |
| IV | 38 | Q9H9J4 | 1 | 1324 | PF00443 | Ubiquitin carboxyl-terminal hydrolase 42 | USP42 |  |
| IV | 38 | Q70EL4 | 1 | 1123 | PF00443 | Ubiquitin carboxyl-terminal hydrolase 43 | USP43 |  |
| IV | 38 | Q9H0E7 | 1 | 712 | PF00443 | Ubiquitin carboxyl-terminal hydrolase 44 | USP44 |  |
| IV | 38 | Q70EL2 | 1 | 814 | PF00443 | Ubiquitin carboxyl-terminal hydrolase 45 | USP45 | Leber congenital amaurosis 19 (LCA19) (MIM:618513) |
| IV | 38 | P62068 | 1 | 366 | PF00443 | Ubiquitin carboxyl-terminal hydrolase 46 | USP46 |  |
| IV | 38 | Q96K76 | 1 | 1375 | PF00443 | Ubiquitin carboxyl-terminal hydrolase 47 | USP47 |  |
| IV | 38 | Q86UV5 | 1 | 1035 | PF00443 | Ubiquitin carboxyl-terminal hydrolase 48 | USP48 USP31 |  |
| IV | 38 | Q70CQ1 | 1 | 688 | PF00443 | Ubiquitin carboxyl-terminal hydrolase 49 | USP49 |  |
| IV | 38 | P45974 | 1 | 858 | PF00443 | Ubiquitin carboxyl-terminal hydrolase 5 | USP5 ISOT |  |
| IV | 38 | Q70EL3 | 0 | 339 | PF00443 | Inactive ubiquitin carboxyl-terminal hydrolase 50 | USP50 |  |
| IV | 38 | Q70EK9 | 1 | 711 | PF00443 | Ubiquitin carboxyl-terminal hydrolase 51 | USP51 |  |
| IV | 38 | Q70EK8 | 0 | 1073 | PF00443 | Inactive ubiquitin carboxyl-terminal hydrolase 53 | USP53 KIAA1350 | Cholestasis, progressive familial intrahepatic, 7, with or without hearing loss (PFIC7) (MIM:619658) |
| IV | 38 | Q70EL1 | 0 | 1684 | PF00443 | Inactive ubiquitin carboxyl-terminal hydrolase 54 | USP54 C10orf29 |  |
| IV | 38 | P35125 | 1 | 1406 | PF00443 | Ubiquitin carboxyl-terminal hydrolase 6 | USP6 HRP1 TRE2 | A chromosomal aberration involving USP6 is a common genetic feature of aneurysmal bone cyst, a benign osseous neoplasm (pmid\|15026324) |
| IV | 38 | Q93009 | 1 | 1102 | PF00443 | Ubiquitin carboxyl-terminal hydrolase 7 | USP7 HAUSP | Hao-Fountain syndrome (HAFOUS) (MIM:616863) |
| IV | 38 | P40818 | 1 | 1118 | PF00443 | Ubiquitin carboxyl-terminal hydrolase 8 | USP8 KIAA0055 UBPY | Pituitary adenoma 4, ACTH-secreting (PITA4) (MIM:219090) |
| IV | 38 | C9JPN9 | 1 | 530 | PF00443 | Ubiquitin carboxyl-terminal hydrolase 17-like protein 12 | USP17L12 |  |
| IV | 38 | Q93008 | 1 | 2554 | PF00443 | Probable ubiquitin carboxyl-terminal hydrolase FAF-X | USP9X DFFRX FAM USP9 | Intellectual developmental disorder, X-linked 99 (XLID99) (MIM:300919); Intellectual developmental disorder, X-linked 99, syndromic, female-restricted (MRXS99F) (MIM:300968) |
| IV | 38 | O00507 | 1 | 2555 | PF00443 | Probable ubiquitin carboxyl-terminal hydrolase FAF-Y | USP9Y DFFRY | Spermatogenic failure Y-linked 2 (SPGFY2) (MIM:415000) |
| IV | 38 | Q5W0Q7 | 1 | 1092 | PF15499 | SUMO-specific isopeptidase USPL1 | USPL1 C13orf22 D13S106 |  |
|  | 40 | Q92560 | 1 | 729 | PF01088 | Ubiquitin carboxyl-terminal hydrolase BAP1 | BAP1 KIAA0272 hucep-6 | Mesothelioma, malignant (MESOM) (MIM:156240) |
|  | 40 | P09936 | 1 | 223 | PF01088 | Ubiquitin carboxyl-terminal hydrolase isozyme L1 | UCHL1 | Parkinson disease 5 (PARK5) (MIM:613643); Spastic paraplegia 79, autosomal recessive (SPG79) (MIM:615491) |
|  | 40 | P15374 | 1 | 230 | PF01088 | Ubiquitin carboxyl-terminal hydrolase isozyme L3 | UCHL3 |  |
|  | 40 | Q9Y5K5 | 1 | 329 | PF01088 | Ubiquitin carboxyl-terminal hydrolase isozyme L5 | UCHL5 UCH37 AD-019 CGI-70 |  |
|  | 53 | Q9H8M7 | 1 | 445 | PF13898 | Ubiquitin carboxyl-terminal hydrolase MINDY-3 | MINDY3 C10orf97 CARP DERP5 FAM188A MSTP126 My042 |  |
|  | 53 | Q4G0A6 | 1 | 757 | PF13898 | Probable ubiquitin carboxyl-terminal hydrolase MINDY-4 | MINDY4 C7orf67 FAM188B |  |
|  | 53 | A8MYZ0 | 0 | 460 | PF13898 | Inactive ubiquitin carboxyl-terminal hydrolase MINDY-4B | MINDY4B C3orf76 FAM188B2 |  |
|  | 54 | Q8N5J2 | 1 | 469 | PF04424 | Ubiquitin carboxyl-terminal hydrolase MINDY-1 | MINDY1 FAM63A KIAA1390 |  |
|  | 54 | Q8NBR6 | 1 | 621 | PF04424 | Ubiquitin carboxyl-terminal hydrolase MINDY-2 | MINDY2 FAM63B KIAA1164 |  |
| V | 62 | Q9NP73 | 1 | 1137 | PF02338 | Putative bifunctional UDP-N-acetylglucosamine transferase and deubiquitinase ALG13 | ALG13 CXorf45 GLT28D1 MDS031 | Developmental and epileptic encephalopathy 36 (DEE36) (MIM:300884) |
| V | 62 | Q5VVQ6 | 1 | 348 | PF02338 | Ubiquitin thioesterase OTU1 | YOD1 DUBA8 HIN7 OTUD2 PRO0907 |  |
| V | 62 | Q7L8S5 | 1 | 288 | PF02338 | OTU domain-containing protein 6A | OTUD6A DUBA2 |  |
| V | 62 | Q8TE49 | 1 | 926 | PF02338 | OTU domain-containing protein 7A | OTUD7A C15orf16 CEZANNE2 OTUD7 |  |
| V | 62 | Q6GQQ9 | 1 | 843 | PF02338 | OTU domain-containing protein 7B | OTUD7B ZA20D1 |  |
| V | 62 | Q5VV17 | 1 | 481 | PF02338 | OTU domain-containing protein 1 | OTUD1 DUBA7 OTDC1 |  |
| V | 62 | Q5T2D3 | 1 | 398 | PF02338 | OTU domain-containing protein 3 | OTUD3 KIAA0459 |  |
| V | 62 | Q01804 | 1 | 1114 | PF02338 | OTU domain-containing protein 4 | OTUD4 HIN-1 KIAA1046 |  |
| V | 62 | Q96G74 | 1 | 571 | PF02338 | OTU domain-containing protein 5 | OTUD5 | Multiple congenital anomalies-neurodevelopmental syndrome, X-linked (MCAND) (MIM:301056) |
| V | 62 | P21580 | 1 | 790 | PF02338 | Tumor necrosis factor alpha-induced protein 3 | TNFAIP3 OTUD7C | Autoinflammatory syndrome, familial, Behcet-like 1 (AIFBL1) (MIM:616744) |
| V | 62 | Q96JH7 | 1 | 1222 | PF02338 | Deubiquitinating protein VCPIP1 | VCPIP1 KIAA1850 VCIP135 |  |
| V | 62 | Q9H8Y1 | 0 | 702 |  | Vertnin | VRTN C14orf115 |  |
| V | 62 | Q9UGI0 | 1 | 708 | PF02338 | Ubiquitin thioesterase ZRANB1 | ZRANB1 TRABID |  |
| V | 63 | Q8N6M0 | 1 | 293 | PF10275 | Deubiquitinase OTUD6B | OTUD6B DUBA5 CGI-77 | Intellectual developmental disorder with dysmorphic facies, seizures, and distal limb anomalies (IDDFSDA) (MIM:617452) |
| V | 63 | Q96FW1 | 1 | 271 | PF10275 | Ubiquitin thioesterase OTUB1 | OTUB1 OTB1 OTU1 HSPC263 |  |
| V | 63 | Q96DC9 | 1 | 234 | PF10275 | Ubiquitin thioesterase OTUB2 | OTUB2 C14orf137 OTB2 OTU2 |  |
| V | 64 | Q96BN8 | 1 | 352 | PF16218 | Ubiquitin thioesterase otulin | OTULIN FAM105B | Autoinflammation, panniculitis, and dermatosis syndrome (AIPDS) (MIM:617099) |
| V | 64 | Q9NUU6 | 0 | 356 | PF16218 | Inactive ubiquitin thioesterase OTULINL | OTULINL FAM105A |  |
|  | 70 | O75503 | 1 | 358 | PF15014 | Ceroid-lipofuscinosis neuronal protein 5 | CLN5 | Ceroid lipofuscinosis, neuronal, 5 (CLN5) (MIM:256731) |
| II | 72 | H3BPM6 | 1 | 223 | PF16044 | MKRN2 opposite strand protein | MKRN2OS C3orf83 MKRN2-AS1 |  |
| II | 74 | Q6ICB0 | 1 | 168 | PF05903 | Desumoylating isopeptidase 1 | DESI1 FAM152B PPPDE2 |  |
| II | 74 | Q9BSY9 | 1 | 194 | PF05903 | Deubiquitinase DESI2 | DESI2 C1orf121 FAM152A PPPDE1 CGI-146 PNAS-4 |  |
| II | 75 | Q9H0R3 | 1 | 208 | PF05608 | Transmembrane protein 222 | TMEM222 C1orf160 | Neurodevelopmental disorder with motor and speech delay and behavioral abnormalities (NEDMOSBA) (MIM:619470) |
| II | 76 | O95237 | 1 | 230 | PF04970 | Lecithin retinol acyltransferase | LRAT | Leber congenital amaurosis 14 (LCA14) (MIM:613341) |
| II | 76 | Q96KN4 | 0 | 292 | PF04970 | Protein LRATD1 | LRATD1 FAM84A NSE1 |  |
| II | 76 | Q96KN1 | 0 | 310 | PF04970 | Protein LRATD2 | LRATD2 BCMP101 FAM84B NSE2 |  |
| II | 76 | Q9HDD0 | 1 | 168 | PF04970 | Phospholipase A and acyltransferase 1 | PLAAT1 HRASLS |  |
| II | 76 | Q9NWW9 | 1 | 162 | PF04970 | Phospholipase A and acyltransferase 2 | PLAAT2 HRASLS2 |  |
| II | 76 | P53816 | 1 | 162 | PF04970 | Phospholipase A and acyltransferase 3 | PLAAT3 HRASLS3 HREV107 PLA2G16 |  |
| II | 76 | Q9UL19 | 1 | 164 | PF04970 | Phospholipase A and acyltransferase 4 | PLAAT4 RARRES3 RIG1 TIG3 |  |
| II | 76 | Q96KN8 | 1 | 279 | PF04970 | Phospholipase A and acyltransferase 5 | PLAAT5 HRASLS5 HRLP5 |  |
| VI | 82 | Q9P0U3 | 1 | 644 | PF02902 | Sentrin-specific protease 1 | SENP1 |  |
| VI | 82 | Q9HC62 | 1 | 589 | PF02902 | Sentrin-specific protease 2 | SENP2 KIAA1331 |  |
| VI | 82 | Q9H4L4 | 1 | 574 | PF02902 | Sentrin-specific protease 3 | SENP3 SSP3 SUSP3 |  |
| VI | 82 | Q96HI0 | 1 | 755 | PF02902 | Sentrin-specific protease 5 | SENP5 FKSG45 |  |
| VI | 82 | Q9GZR1 | 1 | 1112 | PF02902 | Sentrin-specific protease 6 | SENP6 KIAA0797 SSP1 SUSP1 FKSG6 |  |
| VI | 82 | Q9BQF6 | 1 | 1050 | PF02902 | Sentrin-specific protease 7 | SENP7 KIAA1707 SSP2 SUSP2 |  |
| VI | 82 | Q96LD8 | 1 | 212 | PF02902 | Sentrin-specific protease 8 | SENP8 DEN1 NEDP1 PRSC2 FKSG8 |  |
