# Supplementary material for: Identification and classification of papain-like cysteine proteinases
Source: J Biol Chem. 2023 May 8;299(6):104801. doi: 10.1016/j.jbc.2023.104801 (PMC10318531; doi:10.1016/j.jbc.2023.104801)
Supplement: Supporting information [file mmc3.docx]

Identification and Classification of Papain-like Cysteine Proteinases

Fatih Ozhelvaci & Kamil Steczkiewicz

**Supporting Information**

##

[**Details on newly identified proteins and families 2**](#_4j78zo1qcifg)

[BIVM (sp|Q86UB2) 2](#_mvj3ggdh24j)

[DUF1839 (PF08893) 2](#_mawnzb518lzg)

[DUF6005 (PF19468) 3](#_4v5oxy5qvvnl)

[DUF261 (PF03196) 3](#_c6f4cegcla1u)

[C19orf54 (sp|Q5BKX5) 3](#_3qgbi4vdtrhf)

[KOG3639 3](#_ierjyl6xzlmw)

[DUF4846 (PF16138) 4](#_190j8srbzcbd)

[DUF4300 (PF14133) 4](#_ysde5bmw894g)

[C14orf28 (sp|Q4W4Y0) 4](#_dz0ebamvbz5m)

[Pox_P4B (PF03292) 5](#_l8h511gnm6cw)

[DUF1717 (PF05414) 5](#_sw5ku6db2xyh)

[Vertnin (sp|Q9H8Y1) 5](#_i6qqvw2g9nz5)

[DUF2459 (PF09601) 5](#_llefawhol70e)

[DUF4796 (PF16044) 5](#_4whjcyqbsmns)

[DUF778 (PF05608) 5](#_jnjoroa1euea)

[DUF6540 (PF20174) 6](#_8ztih5rdoaje)

[DUF4105 (PF13387) 6](#_lidii9fmv41p)

[DUF6695 (PF20405) 6](#_no6o1vi3cow6)

[DUF3525 (PF12039) 7](#_mh7s1on5m2y6)

[Ac81 (PF05820) 7](#_2h74juqnhc97)

[DUF3750 (PF12570) 7](#_ckpfu5ws8ovu)

[**References 8**](#_7s4s9bu4lm0l)

##

## Details on newly identified proteins and families

### BIVM (sp|Q86UB2)

Basic immunoglobulin-like variable motif-containing protein (BIVM), initially identified in deuterostomes [(1)](https://paperpile.com/c/3Ytzvo/i2hDb) contains a previously undetected papain-like domain remotely homologous to BtrH_N and belonging to cluster I. It retains conserved catalytic triad as well as oxyanion hole residue which suggests a possible enzymatic function. It physically interacts with Lethal(3)malignant brain tumor-like protein 2 (L3MBTL2, regulating gene expression by binding to chromatin), Rhombotin-1 (LMO1, potentially involved in gene regulation), Probable inactive peptidyl-prolyl cis-trans isomerase-like 6 (PPIL6), Sperm protein associated with the nucleus on the X chromosome N2 (SPANXN2), as well as with cytoplasmic E3 ubiquitin ligases: TRAF3, TRIM26 and TRIM41 [(2)](https://paperpile.com/c/3Ytzvo/Fo5aS). However, no details regarding the possible functions of BIVM are available. It has a compact structure with a positively charged active site pocket (Figure S1 A).

### DUF1839 (PF08893)

DUF1839 is a bacterial family found mainly in a-proteobacteria (e.g., *Rhizobium*, *Agrobacterium*, *Methylobacterium*), b-proteobacteria (e.g., *Burkholderia*, *Cupriavidus*, *Caballeronia*) and highGC Gram+ bacteria (e.g., *Rhodococcus*). In proteobacteria, genes coding for DUF1839 proteins are consistently located next to genes encoding Glycoside Hydrolase Family 2 protein (position -1 relative to DUF1839 gene), Amino acid-[acyl-carrier-protein] ligase (position +1), Acyl-CoA/Acyl-ACP dehydrogenase (pos. +2) (β-oxidation cycle of fatty acid degradation [(3)](https://paperpile.com/c/3Ytzvo/PqDoT)) and an Acyl carrier protein (pos. +3). Hence, this family might have a non-peptidase function for fatty acid or phospholipid proteins processing in Bacteria. Although DUF1839 proteins lack a canonical catalytic site, they retain cysteine, threonine/serine, and histidine outlining C(T/S)H active site signature. According to the 3D model of WP_097140493.1 protein belonging to this family, threonine/serine maintains proper orientation and distance against histidine, and further with cysteine, altogether suggesting that this protein might remain catalytically active (Figure S1 B). Additionally, histidine is likely to be stabilized by totally conserved aspartate located in the loop between strands β1 and β2. The substitution of catalytic histidine with another polar residue had already been observed for GP42 transglutaminase where cysteine interacts with asparagine [(4)](https://paperpile.com/c/3Ytzvo/UhE8Y). All family members also retain a unique four-helix bundle located mainly between the α-helix and strand β1'. An interaction between this domain and the β1-β2 loop harboring potentially important aspartate might modulate the activity of these proteins upon substrate binding. Although the modeled protein is clearly negatively charged from all sites, the helical bundle displays patches of positive charge.

### DUF6005 (PF19468)

Bacterial family present mainly in Firmicutes (e.g., *Bacillus*, *Paenibacillus*), a-proteobacteria (e.g., *Paracoccus*, *Methylobacterium*, *Mesorhizobium*, *Ensifer*), seawater g-proteobacteria (e.g., *Marinobacter*, *Alteromonas*). Genes encoding DUF6005 proteins are found in the neighborhood of LysE/ArgO family amino acid transporter (pos. -4), MFS transporter (pos. -3), sugar phosphate isomerase/epimerase (pos. -1), petrobactin biosynthesis protein AsbD (pos. +1), AMP-binding protein (pos. +2), siderophore biosynthesis protein (pos. +3/+4), IucA/IucC family siderophore biosynthesis protein (pos. +3/+4). AsbD contains domains typical of nonribosomal peptide synthetases involved in siderophore biosynthesis in bacteria [(5)](https://paperpile.com/c/3Ytzvo/2kd39). LysE/ArgO exporters control levels of Arg/Lys balance in cells. Therefore, they might take part in the bacterial siderophore biosynthesis pathway. DUF6005 proteins are remotely homologous to DUF1839 and, in contrast to the latter, have a canonical catalytic triad, but still display significant structural similarity, including the additional helical bundle (Figure S1 C). DUF1839 representatives have also clearly more charged surfaces and might function in different biological contexts.

### DUF261 (PF03196)

Members of the DUF261 family are present mostly in bacteria (mainly *Spirochetes*), but also in *Trichomonas vaginalis*. Unlike bacterial homologs, the *Trichomonas* protein contains two SH3 motifs preceding the papain-like domain. Bacterial representatives may occur on plasmids, e.g., *Borreliella mayonii* plasmid lp54 or *Borreliella burgdorferi* plasmid pGr-39_lp52 but the function of these proteins remains unknown. The majority of DUF261 proteins retain the catalytic triad, a bare structural core lacking β4, and a positively charged S3 site (Figure S1 D).

### C19orf54 (sp|Q5BKX5)

C19orf54 belongs to a novel family containing mostly eukaryotic proteins with few representatives from proteobacteria. It has conserved catalytic residues, structural core (Figure S1 E), and is remotely homologous to Peptidase C39, C70, C47, and BtrH_N families. During revising this manuscript Haahr and collaborators described this protein as an actin-maturation protease essential for the proper regulation of actin-specific traits [(6)](https://paperpile.com/c/3Ytzvo/hYpQ).

### KOG3639

Proteins found mainly in animals, but also in proteobacteria. Human homologs include DRC7 (sp|Q8IY82) and CEP76 (sp|Q8TAP6) which retain catalytic triad, as well as CC2D2A (sp|Q9P2K1) and CC2D2B (sp|Q6DHV5) completely devoid of the active site. DRC7 is required for sperm flagellum formation and fertility [(7)](https://paperpile.com/c/3Ytzvo/3JZvd). CEP76 regulates centriole duplication, limiting this process to occur only once per cell cycle [(8)](https://paperpile.com/c/3Ytzvo/IQjvw). The detailed mechanism of action is unknown for DRC7 and CEP76 but, both proteins somehow affect the formation of microtubular polymers and according to the conserved catalytic residues, both seem to be catalytically active papain-like enzymes which imposes the question of whether they function as specific peptidases. DRC7 has a big, C-terminal, long β-domain similar to the *Danio rerio* CPAP TCP domain [(9)](https://paperpile.com/c/3Ytzvo/mCldS) (Figure S1 F). Like CPAP TCP, it contains several repeats, and its edge is also lined with aromatic residues important for interactions with other centriolar proteins [(9)](https://paperpile.com/c/3Ytzvo/mCldS). CEP76 in turn has an N-terminal domain highly similar to CEP120 centrosomal protein (pdb|4icx), and a C-terminal domain remotely homologous to *Borrelia Burgdorferi* Bb0689 [(10)](https://paperpile.com/c/3Ytzvo/he5DJ) from CAP Cysteine-rich secretory protein family (PF00188) (Figure S1 G). The CAP family includes 15 human proteins, e.g. AEGL (CRISP1, sp|P54107) potentially playing a role in sperm-egg fusion [(11)](https://paperpile.com/c/3Ytzvo/ya1hz) and related to male infertility [(12)](https://paperpile.com/c/3Ytzvo/lRuUe). Hence, the potential enzymatic function of DRC7 and CEP76 might modulate centriole dynamics by modifying particular centriolar proteins - KOG3639 belongs to cluster III, which contains mostly acetyltransferases, deamidases, detyrosinases, transglutaminases, involved in, e.g., post-translational protein modifications or protein degradation. CC2D2A, encoded by one of the Meckel syndrome-related genes, is critical for cilia formation and might participate in Ca^2+^-regulated signaling pathways (see references in [(13)](https://paperpile.com/c/3Ytzvo/WD1Nh)). Both CC2D2A and CC2D2B contain an N-terminal CEP120-like domain and two CAP-like domains in their C-terminal part (Figure S1 H, I).

### DUF4846 (PF16138)

Proteins belonging to this family are found mainly in CFB group bacteria (e.g., *Chryseobacterium*, *Bacteroidetes*, *Hymenobacter*, *Flavobacteriales*, *Prevotella*), firmicutes (e.g., *Clostridiales*, *Lachnospiraceae*, *Ruminococcus*, *Paenibacillus*), d-proteobacteria (e.g., *Myxococcales*). They have conserved catalytic residues, as well as the "GD" motif characteristic for NlpC/p60 proteins involved in cell wall degradation/modification (Figure S1 J).

### DUF4300 (PF14133)

Bacterial families are present mostly in Firmicutes and CFB group bacteria in the genomic neighborhood of IS-like transposases and S24 family peptidases. According to the conserved structural core and catalytic triad, these proteins might be catalytically active, with the active site pocket lined with negatively charged patches (Figure S1 K). The detailed function of DUF4300 proteins remains unknown.

### C14orf28 (sp|Q4W4Y0)

Known also as dopamine receptor-interacting protein (DRIP1 [(14)](https://paperpile.com/c/3Ytzvo/Rkb9L)) is an uncharacterized human protein related to cluster IV Ubiquitin carboxyl-terminal hydrolases (UCH). It contains a papain-like deubiquitinase domain with catalytic residues and a "fingers" motif for ubiquitin or ubiquitin-like substrate stabilization. Moreover, C14orf28 has additional "fingers"-like β-sheet on the opposite side of the structure which might shape the substrate specificity (Figure S1 L). Its expression levels are altered in colorectal cancer [(15)](https://paperpile.com/c/3Ytzvo/mRZMp), bipolar disorder, and schizophrenia [(16)](https://paperpile.com/c/3Ytzvo/8t0LP) but the details of its molecular function are missing.

### Pox_P4B (PF03292)

Family of viral core proteins [(17)](https://paperpile.com/c/3Ytzvo/wGquh) of unknown function found in *Poxviridae* and *Mimiviridae*. P4B proteins are catalytically inactive and only one known member of the family, p4b precursor from *Pteropox virus* (YP_009268812.1) retains a full catalytic site (Figure S1 M). They are remotely similar to deubiquitinases (e.g., human USP12, USP35, USP21) from cluster IV, which already contains multiple viral proteins with deubiquitinating activity.

### DUF1717 (PF05414)

Protein family present in viruses, e.g., Apple stem grooving virus also known as Citrus tatter leaf virus. In viral POLG protein (sp|P36309) co-occurs with a methyltransferase, helicase, RNA-dependent RNA polymerase, and a coat protein. DUF1717 proteins retain a very reduced papain-like domain harboring catalytic dyad (Figure S1 N) and belong to cluster V with other viral peptidases.

### Vertnin (sp|Q9H8Y1)

Vertnin (VRTN, C14orf115) VRTN is a DNA-binding transcription factor and its variants are associated with a varying number of thoracic vertebrae in pigs from different populations [(18, 19)](https://paperpile.com/c/3Ytzvo/ciVa5+N6nU3). It contains a newly detected OTU-like domain, enzymatically inactive due to the lack of all catalytic residues, followed by five positively charged HTH domains not belonging to any protein family. The role of the OTU-like domain remains unknown. Its highly reliable AlphaFold2 model shows reduced strand β3 encoded before the α-helix, β1' and β5 having reversed direction, and β4 missing (Figure S1 O), which makes it one the most diverged representatives of papain-like proteins.

### DUF2459 (PF09601)

Bacterial family of unknown function. In *Rhizobium* and *Agrobacterium* genes coding for DUF2459 proteins are located within urease gene clusters which might suggest it is one of yet uncharacterized urease-related pathway members. The 3D model of *Leptolyngbyaceae cyanobacterium* protein (NJP09016.1) shows an α-helical insertion between strands β4 and β5 which reduces the size of the active site pocket (Figure S1 P).

### DUF4796 (PF16044)

The domain of unknown function with conserved catalytic residues (Figure S1 Q), present in animals including flatworms, nematodes, insects, reptiles, marsupials, and mammals. Human representative, MKRN2OS (C3orf83, MKRN2 opposite strand protein) is expressed mainly in mucus-secreting cells and localizes to Golgi apparatus [(20)](https://paperpile.com/c/3Ytzvo/sfe7V) yet its function remains unknown.

### DUF778 (PF05608)

Eukaryotic family of transmembrane proteins. Its members include *Arabidopsis thaliana* RTE1 (Protein Reversion to ethylene sensitivity 1) - a negative regulator of the ethylene signaling pathway [(21, 22)](https://paperpile.com/c/3Ytzvo/0xYVR+dBJzv) where it promotes conformational changes of ETR1 via an unknown mechanism [(23)](https://paperpile.com/c/3Ytzvo/DJ8h4), and human TMEM222 (Transmembrane protein 222, C1orf160); for both proteins detailed function remain unknown. Although TMEM222 is expressed in all tissues, its RNA levels are predominantly increased in the brain; it localizes to the plasma membrane and cytosol, but may also be found in cell junctions [(20)](https://paperpile.com/c/3Ytzvo/sfe7V). Deleterious variants in TMEM222 are likely to cause neurodevelopmental disorders and play roles in post-synaptic vesicles in neurons [(24)](https://paperpile.com/c/3Ytzvo/7606e). DUF778 proteins retain conserved catalytic residues, have permuted α-helix followed by three transmembrane α-helices (Figure S1 R), and belong to cluster II together with desumolyases, acetyltransferases, and phospholipases. They might affect the functioning of membrane receptors either by reshaping the membrane environment or participating in other protein modifications.

### DUF6540 (PF20174)

Fungal family (mostly filamentous *Ascomycota* and mushroom-forming *Basidiomycota*) of uncharacterized proteins. These proteins retain a catalytic triad, have permuted α-helix, and probably unstructured ~20aa loop directly preceding α-helix and possibly regulating access to the active site (Figure S1 S).

### DUF4105 (PF13387)

Bacterial family of unknown function present predominantly in Proteobacteria. In e-proteobacteria are localized in the neighborhood of protein translocase subunit SecF (pos. -4), protein translocase subunit SecD (pos. -3), Apolipoprotein N-acyltransferase (pos. -1), DUF3015 family protein (pos. +1), whereas in g-proteobacteria TerC family protein (pos. -5, associated with resistance to Tellurium [(25)](https://paperpile.com/c/3Ytzvo/ohPAo)), CitMHS family transporter (pos. -4), DUF2388 domain-containing protein (pos. -3), AEC family transporter (pos. -2), GFA family protein (pos. -1, Glutathione-dependent formaldehyde-activating enzyme in formaldehyde detoxification [(26)](https://paperpile.com/c/3Ytzvo/o5Bxf)), DUF3015 domain-containing protein (pos. +1), DUF2388 domain-containing protein (pos. +2/+3), DUF1127 domain-containing protein (pos. +4, potentially associated with alkali resistance [(27)](https://paperpile.com/c/3Ytzvo/XWyrW)). The detailed function of DUF4105 proteins remains unknown, but their co-occurrence with many transmembrane proteins, including translocases and resistance-related transporters, as well as its predicted evolutionary relationship to cluster II families, might suggest functions related to membrane proteins/lipids processing. The catalytic site of the papain-like domain is buried in a deep pocket (Figure S1 T).

### DUF6695 (PF20405)

Bacterial family of proteins of unknown function, closely related to Type VI secretion system effector, TseH from *Vibrio cholerae* (pdb|6v98 [(28)](https://paperpile.com/c/3Ytzvo/0tgCM)). Genes encoding DUF6695 proteins localize in genomes together with Threonine-tRNA ligase (pos. -2), PAAR domain-containing protein (pos. +1). The PAAR protein is required for delivering bacterial effectors and VgrG and limits the transport of alternative phospholipase effectors. ThrRS inhibits translation by preventing the ribosome from binding to mRNA, whereas tRNA(Thr) functions as an antirepressor, allowing for fine-grained regulation of enzyme production [(29)](https://paperpile.com/c/3Ytzvo/8QRCc). Structural model of DUF6695 representative, hypothetical protein from *Seonamhaeicola marinus* (WP_148540441.1) has additional, conserved C-terminal domain composed of 6-7 β-strands. This domain displays no sequence similarity to any known protein structure, however, DALI was able to find its resemblance to WD40 blades from the BPB domain of yeast's tRNA binding Trn734 (pdb|6jp6-A [(30)](https://paperpile.com/c/3Ytzvo/My7KB), Z-score 4.5) and GID4 from the glucose-induced degradation complex GID (pdb|7wug-4 [(31)](https://paperpile.com/c/3Ytzvo/RlrYy), Z-score 4.3) (Figure S1 U). Taken together, DUF6695 proteins might function as one of the toxic effector proteins interfering with the host's tRNA processing machinery or protein degradation pathways.

### DUF3525 (PF12039)

Potential peptidases from hypoviruses attacking fungi. They are present in POLB polyprotein together with Peptidase C8 or Peptidase C7, RNA-dependent RNA polymerase, and helicase domains. DUF3525 has a conserved catalytic triad and compact papain-like structure (Figure S1 V).

### Ac81 (PF05820)

Viral transmembrane proteins, e.g., baculoviral AC81 essential for the assembly of nucleocapsids with envelopes through yet unknown mechanisms [(32)](https://paperpile.com/c/3Ytzvo/PdURI). Members of the family have conserved catalytic residues, short and permuted α-helix followed by two transmembrane helices (Figure S1 W), and might function as peptidases essential for viral development.

### DUF3750 (PF12570)

Bacterial family of unknown function. Despite retaining histidine, glutamate, and oxyanion hole tyrosine, DUF3750 proteins lack catalytic cysteine and have serine instead. The active site pocket is positively charged and the papain-like domain is followed by a non-polar, 6-stranded β-sheet (Figure S1 X).

##

## References

[1. Yoder,J.A., Hawke,N.A., Eason,D.D., Mueller,M.G., Davids,B.J., Gillin,F.D. and Litman,G.W. (2002) BIVM, a novel gene widely distributed among deuterostomes, shares a core sequence with an unusual gene in Giardia lamblia. *Genomics*, **79**, 750–755.](http://paperpile.com/b/3Ytzvo/i2hDb)

[2. Orchard,S., Ammari,M., Aranda,B., Breuza,L., Briganti,L., Broackes-Carter,F., Campbell,N.H., Chavali,G., Chen,C., del-Toro,N., *et al.* (2014) The MIntAct project--IntAct as a common curation platform for 11 molecular interaction databases. *Nucleic Acids Res.*, **42**, D358–63.](http://paperpile.com/b/3Ytzvo/Fo5aS)

[3. Campbell,J.W. and Cronan,J.E.,Jr (2002) The enigmatic Escherichia coli fadE gene is yafH. *J. Bacteriol.*, **184**, 3759–3764.](http://paperpile.com/b/3Ytzvo/PqDoT)

[4. Reiss,K., Kirchner,E., Gijzen,M., Zocher,G., Löffelhardt,B., Nürnberger,T., Stehle,T. and Brunner,F. (2011) Structural and phylogenetic analyses of the GP42 transglutaminase from Phytophthora sojae reveal an evolutionary relationship between oomycetes and marine Vibrio bacteria. *J. Biol. Chem.*, **286**, 42585–42593.](http://paperpile.com/b/3Ytzvo/UhE8Y)

[5. Najimi,M., Lemos,M.L. and Osorio,C.R. (2008) Identification of siderophore biosynthesis genes essential for growth of Aeromonas salmonicida under iron limitation conditions. *Appl. Environ. Microbiol.*, **74**, 2341–2348.](http://paperpile.com/b/3Ytzvo/2kd39)

[6. Haahr,P., Galli,R.A., van den Hengel,L.G., Bleijerveld,O.B., Kazokaitė-Adomaitienė,J., Song,J.-Y., Kroese,L.J., Krimpenfort,P., Baltissen,M.P., Vermeulen,M., *et al.* (2022) Actin maturation requires the ACTMAP/C19orf54 protease. *Science*, **377**, 1533–1537.](http://paperpile.com/b/3Ytzvo/hYpQ)

[7. Morohoshi,A., Miyata,H., Shimada,K., Nozawa,K., Matsumura,T., Yanase,R., Shiba,K., Inaba,K. and Ikawa,M. (2020) Nexin-Dynein regulatory complex component DRC7 but not FBXL13 is required for sperm flagellum formation and male fertility in mice. *PLoS Genet.*, **16**, e1008585.](http://paperpile.com/b/3Ytzvo/3JZvd)

[8. Tsang,W.Y., Spektor,A., Vijayakumar,S., Bista,B.R., Li,J., Sanchez,I., Duensing,S. and Dynlacht,B.D. (2009) Cep76, a centrosomal protein that specifically restrains centriole reduplication. *Dev. Cell*, **16**, 649–660.](http://paperpile.com/b/3Ytzvo/IQjvw)

[9. Cottee,M.A., Muschalik,N., Wong,Y.L., Johnson,C.M., Johnson,S., Andreeva,A., Oegema,K., Lea,S.M., Raff,J.W. and van Breugel,M. (2013) Crystal structures of the CPAP/STIL complex reveal its role in centriole assembly and human microcephaly. *Elife*, **2**, e01071.](http://paperpile.com/b/3Ytzvo/mCldS)

[10. Brangulis,K., Jaudzems,K., Petrovskis,I., Akopjana,I., Kazaks,A. and Tars,K. (2015) Structural and functional analysis of BB0689 from Borrelia burgdorferi, a member of the bacterial CAP superfamily. *J. Struct. Biol.*, **192**, 320–330.](http://paperpile.com/b/3Ytzvo/he5DJ)

[11. Hayashi,M., Fujimoto,S., Takano,H., Ushiki,T., Abe,K., Ishikura,H., Yoshida,M.C., Kirchhoff,C., Ishibashi,T. and Kasahara,M. (1996) Characterization of a human glycoprotein with a potential role in sperm-egg fusion: cDNA cloning, immunohistochemical localization, and chromosomal assignment of the gene (AEGL1). *Genomics*, **32**, 367–374.](http://paperpile.com/b/3Ytzvo/ya1hz)

[12. Mou,L. and Xie,N. (2017) Male infertility-related molecules involved in sperm-oocyte fusion. *J. Reprod. Dev.*, **63**, 1–7.](http://paperpile.com/b/3Ytzvo/lRuUe)

[13. Tallila,J., Jakkula,E., Peltonen,L., Salonen,R. and Kestilä,M. (2008) Identification of CC2D2A as a Meckel syndrome gene adds an important piece to the ciliopathy puzzle. *Am. J. Hum. Genet.*, **82**, 1361–1367.](http://paperpile.com/b/3Ytzvo/WD1Nh)

[14. Feng,G., Hannan,F., Reale,V., Hon,Y.Y., Kousky,C.T., Evans,P.D. and Hall,L.M. (1996) Cloning and functional characterization of a novel dopamine receptor from Drosophila melanogaster. *J. Neurosci.*, **16**, 3925–3933.](http://paperpile.com/b/3Ytzvo/Rkb9L)

[15. Yang,X., Hu,Y., Liu,Y., Liu,W., Zhao,X., Liu,M. and Tang,H. (2017) C14orf28 downregulated by miR-519d contributes to oncogenicity and regulates apoptosis and EMT in colorectal cancer. *Mol. Cell. Biochem.*, **434**, 197–208.](http://paperpile.com/b/3Ytzvo/mRZMp)

[16. Zhan,L., Kerr,J.R., Lafuente,M.-J., Maclean,A., Chibalina,M.V., Liu,B., Burke,B., Bevan,S. and Nasir,J. (2011) Altered expression and coregulation of dopamine signalling genes in schizophrenia and bipolar disorder. *Neuropathol. Appl. Neurobiol.*, **37**, 206–219.](http://paperpile.com/b/3Ytzvo/8t0LP)

[17. Vanslyke,J.K. and Hruby,D.E. (1994) Immunolocalization of vaccinia virus structural proteins during virion formation. *Virology*, **198**, 624–635.](http://paperpile.com/b/3Ytzvo/wGquh)

[18. Yang,J., Huang,L., Yang,M., Fan,Y., Li,L., Fang,S., Deng,W., Cui,L., Zhang,Z., Ai,H., *et al.* (2016) Possible introgression of the VRTN mutation increasing vertebral number, carcass length and teat number from Chinese pigs into European pigs. *Sci. Rep.*, **6**, 19240.](http://paperpile.com/b/3Ytzvo/ciVa5)

[19. Liu,Q., Yue,J., Niu,N., Liu,X., Yan,H., Zhao,F., Hou,X., Gao,H., Shi,L., Wang,L., *et al.* (2020) Genome-Wide Association Analysis Identified BMPR1A as a Novel Candidate Gene Affecting the Number of Thoracic Vertebrae in a Large White × Minzhu Intercross Pig Population. *Animals (Basel)*, **10**.](http://paperpile.com/b/3Ytzvo/N6nU3)

[20. Sjöstedt,E., Zhong,W., Fagerberg,L., Karlsson,M., Mitsios,N., Adori,C., Oksvold,P., Edfors,F., Limiszewska,A., Hikmet,F., *et al.* (2020) An atlas of the protein-coding genes in the human, pig, and mouse brain. *Science*, **367**.](http://paperpile.com/b/3Ytzvo/sfe7V)

[21. Resnick,J.S., Wen,C.-K., Shockey,J.A. and Chang,C. (2006) REVERSION-TO-ETHYLENE SENSITIVITY1, a conserved gene that regulates ethylene receptor function in Arabidopsis. *Proc. Natl. Acad. Sci. U. S. A.*, **103**, 7917–7922.](http://paperpile.com/b/3Ytzvo/0xYVR)

[22. Binder,B.M. (2020) Ethylene signaling in plants. *J. Biol. Chem.*, **295**, 7710–7725.](http://paperpile.com/b/3Ytzvo/dBJzv)

[23. Resnick,J.S., Rivarola,M. and Chang,C. (2008) Involvement of RTE1 in conformational changes promoting ETR1 ethylene receptor signaling in Arabidopsis. *Plant J.*, **56**, 423–431.](http://paperpile.com/b/3Ytzvo/DJ8h4)

[24. Polla,D.L., Farazi Fard,M.A., Tabatabaei,Z., Habibzadeh,P., Levchenko,O.A., Nikuei,P., Makrythanasis,P., Hussain,M., von Hardenberg,S., Zeinali,S., *et al.* (2021) Biallelic variants in TMEM222 cause a new autosomal recessive neurodevelopmental disorder. *Genet. Med.*, **23**, 1246–1254.](http://paperpile.com/b/3Ytzvo/7606e)

[25. Kormutakova,R., Klucar,L. and Turna,J. (2000) DNA sequence analysis of the tellurite-resistance determinant from clinical strain of Escherichia coli and identification of essential genes. *Biometals*, **13**, 135–139.](http://paperpile.com/b/3Ytzvo/ohPAo)

[26. Goenrich,M., Bartoschek,S., Hagemeier,C.H., Griesinger,C. and Vorholt,J.A. (2002) A glutathione-dependent formaldehyde-activating enzyme (Gfa) from Paracoccus denitrificans detected and purified via two-dimensional proton exchange NMR spectroscopy. *J. Biol. Chem.*, **277**, 3069–3072.](http://paperpile.com/b/3Ytzvo/o5Bxf)

[27. Ji,Z.J., Wu,Z.Y., Chen,W.F., Wang,E.T., Yan,H., Cui,Q.G., Zhang,J.X., Wang,L. and Ma,S.J. (2020) Physiological and symbiotic variation of a long-term evolved Rhizobium strain under alkaline condition. *Syst. Appl. Microbiol.*, **43**, 126125.](http://paperpile.com/b/3Ytzvo/XWyrW)

[28. Hersch,S.J., Watanabe,N., Stietz,M.S., Manera,K., Kamal,F., Burkinshaw,B., Lam,L., Pun,A., Li,M., Savchenko,A., *et al.* (2020) Envelope stress responses defend against type six secretion system attacks independently of immunity proteins. *Nat Microbiol*, **5**, 706–714.](http://paperpile.com/b/3Ytzvo/0tgCM)

[29. Moine,H., Romby,P., Springer,M., Grunberg-Manago,M., Ebel,J.P., Ehresmann,B. and Ehresmann,C. (1990) Escherichia coli threonyl-tRNA synthetase and tRNA(Thr) modulate the binding of the ribosome to the translational initiation site of the thrS mRNA. *J. Mol. Biol.*, **216**, 299–310.](http://paperpile.com/b/3Ytzvo/8QRCc)

[30. Hirata,A., Okada,K., Yoshii,K., Shiraishi,H., Saijo,S., Yonezawa,K., Shimizu,N. and Hori,H. (2019) Structure of tRNA methyltransferase complex of Trm7 and Trm734 reveals a novel binding interface for tRNA recognition. *Nucleic Acids Res.*, **47**, 10942–10955.](http://paperpile.com/b/3Ytzvo/My7KB)

[31. Qiao,S., Lee,C.-W., Sherpa,D., Chrustowicz,J., Cheng,J., Duennebacke,M., Steigenberger,B., Karayel,O., Vu,D.T., von Gronau,S., *et al.* (2022) Cryo-EM structures of Gid12-bound GID E3 reveal steric blockade as a mechanism inhibiting substrate ubiquitylation. *Nat. Commun.*, **13**, 3041.](http://paperpile.com/b/3Ytzvo/RlrYy)

[32. Dong,F., Wang,J., Deng,R. and Wang,X. (2016) Autographa californica multiple nucleopolyhedrovirus gene ac81 is required for nucleocapsid envelopment. *Virus Res.*, **221**, 47–57.](http://paperpile.com/b/3Ytzvo/PdURI)
