# Supplementary figures and images for: Identification and classification of papain-like cysteine proteinases
Source: J Biol Chem. 2023 May 8;299(6):104801. doi: 10.1016/j.jbc.2023.104801 (PMC10318531; doi:10.1016/j.jbc.2023.104801)

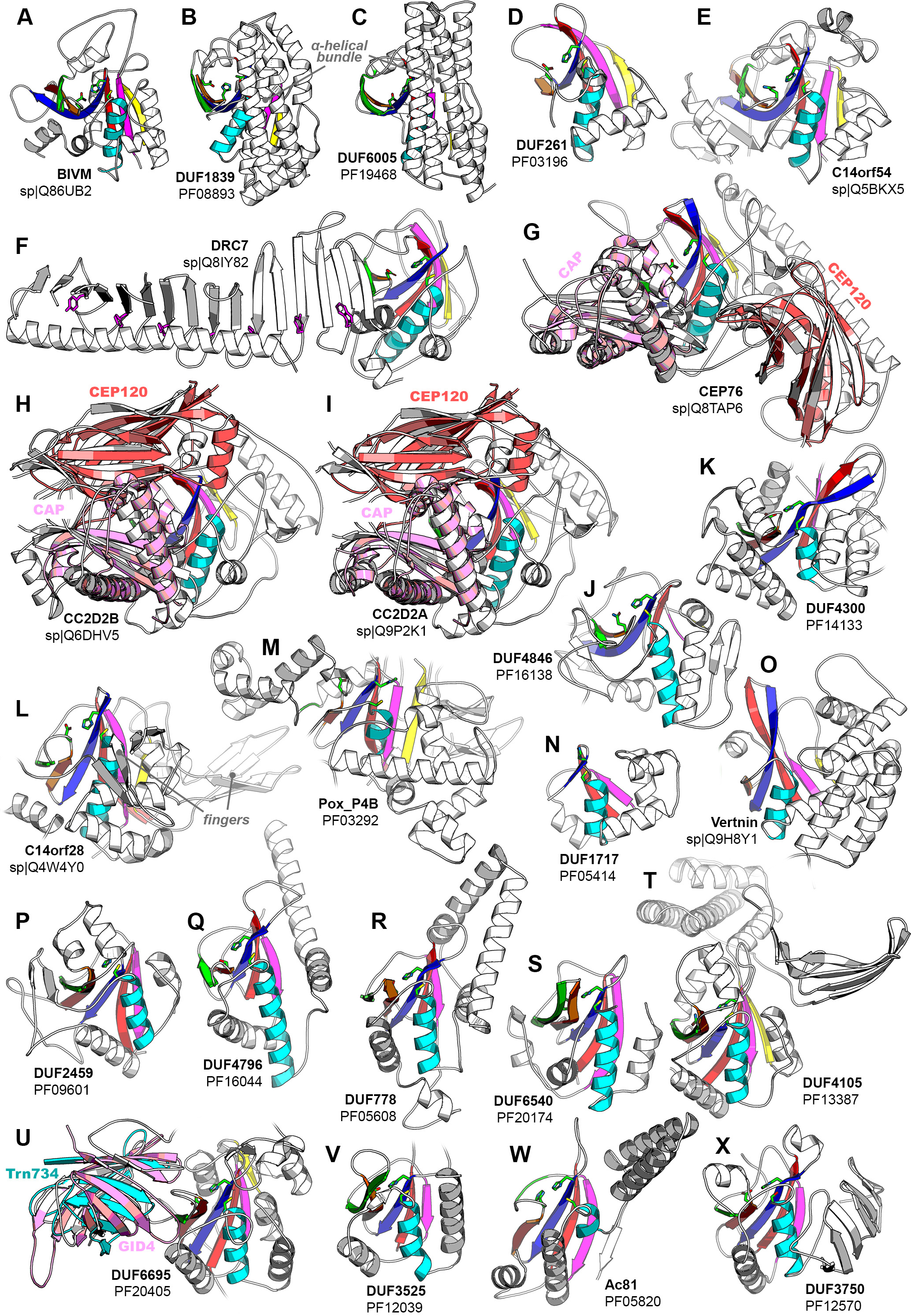

Supplement: Figure S1 [file figs1.jpg]
